# Assessing the suitability for *Aedes albopictus* and dengue transmission risk in China with a delay differential equation model

**DOI:** 10.1371/journal.pntd.0009153

**Published:** 2021-03-26

**Authors:** Soeren Metelmann, Xiaobo Liu, Liang Lu, Cyril Caminade, Keke Liu, Lina Cao, Jolyon M. Medlock, Matthew Baylis, Andrew P. Morse, Qiyong Liu

**Affiliations:** 1 Institute for Infection and Global Health, University of Liverpool, Liverpool, United Kingdom; 2 NIHR Health Protection Research Unit in Emerging and Zoonotic Infections, Liverpool, United Kingdom; 3 State Key Laboratory of Infectious Disease Prevention and Control, Collaborative Innovation Center for Diagnosis and Treatment of Infectious Diseases, WHO Collaborating Centre for Vector Surveillance and Management, National Institute for Communicable Disease Control and Prevention, Chinese Center for Disease Control and Prevention, Beijing, China; 4 School of Public Health, Shandong University, Jinan, China; 5 Medical Entomology Group, Public Health England, Salisbury, United Kingdom; 6 School of Environmental Sciences, University of Liverpool, Liverpool, United Kingdom; Universite de Montreal, CANADA

## Abstract

Dengue is considered non-endemic to mainland China. However, travellers frequently import the virus from overseas and local mosquito species can then spread the disease in the population. As a consequence, mainland China still experiences large dengue outbreaks. Temperature plays a key role in these outbreaks: it affects the development and survival of the vector and the replication rate of the virus. To better understand its implication in the transmission risk of dengue, we developed a delay differential equation model that explicitly simulates temperature-dependent development periods and tested it with collected field data for the Asian tiger mosquito, *Aedes albopictus*. The model predicts mosquito occurrence locations with a high accuracy (Cohen’s κ of 0.78) and realistically replicates mosquito population dynamics. Analysing the infection dynamics during the 2014 dengue outbreak that occurred in Guangzhou showed that the outbreak could have lasted for another four weeks if mosquito control interventions had not been undertaken. Finally, we analyse the dengue transmission risk in mainland China. We find that southern China, including Guangzhou, can have more than seven months of dengue transmission per year while even Beijing, in the temperate north, can have dengue transmission during hot summer months. The results demonstrate the importance of using detailed vector and infection ecology, especially when vector-borne disease transmission risk is modelled over a broad range of climatic zones.

## Author summary

Dengue is a mosquito-borne disease and the transmission of its virus depends on four factors: The presence of 1) the virus, 2) the human host, 3) the mosquito vector, and 4) the suitability of environmental conditions. Mainland China faces regular dengue outbreaks. Because the virus is constantly imported by infected travellers into the susceptible populations of Chinese metropolises, factors 1) and 2) are almost always met. We now investigate factors 3) and 4) to see when and where the risk for outbreaks is greatest. We use a novel model that considers the development of both the mosquitoes and the viruses under different climate conditions. Our findings suggest that the Asian tiger mosquito (*Aedes albopictus*) can potentially transmit dengue virus over large parts of mainland China; the biggest transmission risk is simulated over southern China where large outbreaks have occurred historically, but also over the temperate north, as far as Beijing, where smaller dengue outbreaks could occur during hot summer months.

## Introduction

Mosquito-borne diseases have long been an issue in mainland China (People’s Republic of China) [1]. Arboviruses are numerous [2], malaria cases often reached 20 to 30 million cases in a single year during the last century [3,4] and southern China was a hotspot for filarial nematode transmission [5]. But while malaria has been nearly and lymphatic filariasis completely eliminated during this century [6,4], thanks to intensified control measures, arboviruses such as dengue still persist and cause outbreaks with the biggest burden reported for Yunnan, the province bordering Vietnam, Laos and Myanmar, and Guangdong on the south coast of China [2].

Dengue cases were only sporadically identified in China in the early 20^th^ century [7]. This changed in 1978, when a dengue outbreak with about 22,000 cases occurred in Guangdong province [8]. Since that year, cases have been reappearing every year, frequently introduced by travellers from dengue-endemic regions [2]. In some years, introduced infections lead to local transmission resulting in large dengue outbreaks: roughly 500,000 cases were reported in 1980, 100,000 cases in 1986 and nearly 50,000 cases in 2014 [8,9]. The city of Guangzhou, with 15 million inhabitants, was the epicentre of the 2014 outbreak that soon spread to the whole province of Guangdong [10]. Unlike in many other dengue-ridden countries, the main vector of dengue in Guangzhou is not *Ae*. *aegypti*, the yellow fever mosquito which is absent in Guangzhou, but the less efficient dengue vector *Ae*. *albopictus*, the Asian tiger mosquito, long since established over large parts of China [11,12].

Numerous studies have investigated the 2014 dengue outbreak, looking for example at the impacts of early year precipitation [13] and resulting surface water for breeding sites [14], at the impacts of vaccination and control [15], initial case importation and interventions [13,16] or human mobility [10,17]. These studies use dynamical models based on ordinary differential equations which are useful tools to describe vector-host population and infection dynamics. However, the description of delayed temporal effects by ordinary differential equation (ODE) models is not very accurate. The pathogen incubation period in the mosquito represents such a delayed effect [18] and the use of ODEs can lead to an overestimation of transmission activity. Delayed differential equations (DDEs) can model these effects more accurately because their rate of change depends not only on the current but also on previous time steps. A temperature-dependent incubation rate for example, can be modelled depending on the whole temperature development since the start of the infection and not only on the current day temperature.

DDE models were introduced to the field of entomology by the work of Gurney et al. on blowflies and by Nisbet and Gurney for the general theory [19,20]. Even though their work was quickly followed by a first DDE model for the mosquito *Ae*. *aegypti* [21], DDE models were not as frequently used as ODE models because they are more complex to solve. But there is an increasing number of published studies using DDE models during more recent years; these models have been used to study mosquito population dynamics [22,23] and the transmission of a range of mosquito-borne diseases, including West Nile [24], Rift Valley fever [25], chikungunya [26] and malaria [27].

Here, we develop a novel DDE model to simulate mosquito dynamics in mainland China, as well as dengue transmission dynamics during the 2014 outbreak. We then analyse the potential dengue season length for mainland China and highlight four highly populated cities at risk.

## Model and methods

We use the model developed for Europe and the UK in [28] as a framework to describe the vector population dynamics. This model used ordinary differential equations (ODEs), and classes of eggs, (aquatic) juveniles, immature females, mature females, and special diapausing eggs, to describe all life stages. In order to introduce virus transmission in the model, we now add dengue-infected female mosquitoes to the equation system along with human hosts. Also, the transitions between vector classes are modelled using maturation delays, in order to describe the infection dynamics more realistically. The resulting delayed differential equation (DDE) model comprises seven equations for the vector and four equations for the human host. The derivation of the DDE system from the original ODEs is explained further in S1.1 in S1 Supplementary Material.

### Vector dynamics

**Fig 1:**
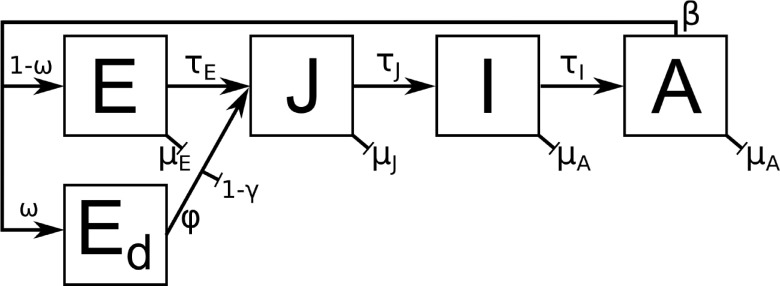
Life stages of *Aedes albopictus*. The cycle goes from eggs, E, to larvae/pupae (juvenile), J, to newly eclosed (immature) females, I, to mature adult females, A. Adult female mosquitoes lay normal eggs, E, in the summer months or diapausing eggs, E_d_, at the end of the season. Diapausing eggs overwinter and are activated by warmer and longer days in spring.

The vector life cycle is shown in Fig 1. Eggs, E, hatch after a certain development time. The emerging larvae and pupae are combined in an aquatic juvenile class, J. After a certain time, juveniles develop into 50% newly eclosed female adults, I; male adults, which play no role in virus transmission, are ignored. After a resting stage, females mature to the adult stage A, take their first blood meal and start to lay eggs. Normal eggs, E, are laid in spring and summer, while diapausing eggs, E_d_, are laid in autumn when the days are getting shorter. These eggs stay inactive over the winter and are activated by warmer and longer days in spring. All classes experience some temperature-dependent mortalities. Only juveniles also experience a density-dependent mortality based on an environmental capacity K which relies on rainfall and human population density.

The transitions and mortalities of classes are now described by a system of delay differential equations. The structure of a DDE can be described by a recruitment term R with all incoming individuals from other classes, a maturation term M with the individuals that change to another class, and a mortality term for the individuals that leave the system. With parameter definitions given in Table 1, the population dynamics in the vector are described by:

$$\begin{array}{c} \frac{d}{dt}E\left(t\right)=\beta \left(1-\omega \right)A\left(t\right)-\beta \left(1-\omega \right)A\left(t-\tau_{E}\right)exp\left(-\int_{t-\tau_{e}}^{t}\mu_{E}\left(\sigma \right)d\sigma \right)-\mu_{E}E\left(t\right)\\ =R_{E}(t)-M_{E}(t)-\mu_{E}E(t) \end{array}$$ (1)

$$\frac{d}{dt}E_{d}\left(t\right)=\beta \omega A\left(t\right)-\varphi E_{d}\left(t\right)$$ (2)

$$\begin{array}{c} \frac{d}{dt}J\left(t\right)=M_{E}\left(t\right)+\varphi \gamma E_{d}\left(t\right)-\frac{\left(M_{E}\left(t-\tau_{J}\right)+\varphi \gamma E_{d}\left(t-\tau_{J}\right)\right)exp\left(-\int_{t-\tau_{J}}^{t}\mu_{J}\left(\sigma \right)d\sigma \right)}{1+\left(M_{E}\left(t-\tau_{J}\right)+\varphi \gamma E_{d}\left(t-\tau_{J}\right)\right)\int_{t-\tau_{e}}^{t}L\left(u\right)du}-\mu_{J}J\left(t\right)-\frac{J{\left(t\right)}^{2}}{K\left(t\right)}\\ =R_{J}(t)-M_{J}(t)-\mu_{J}J(t)-\frac{J{\left(t\right)}^{2}}{K(t)} \end{array}$$ (3)

$$\begin{array}{c} \frac{d}{dt}I\left(t\right)=\frac{1}{2}M_{J}\left(t\right)-\frac{1}{2}M_{J}\left(t-\tau_{I}\right)exp\left(-\int_{t-\tau_{I}}^{t}\mu_{A}\left(\sigma \right)d\sigma \right)-\mu_{A}I\left(t\right)\\ =R_{I}(t)-M_{I}(t)-\mu_{A}I(t) \end{array}$$ (4)

$$\frac{d}{dt}A\left(t\right)=M_{I}\left(t\right)-\mu_{A}A\left(t\right)$$ (5)

$$with\;L(u)=\frac{1}{K(u)}exp\left(\int_{u-\tau_{J}}^{u}\mu_{J}(\sigma )d\sigma \right).$$

Mosquito maturation periods, τ, and mortality rates, μ, depend on daily mean temperature T. Only the egg hatching rate in spring is triggered by the average temperature over the past seven days, T_7_. The winter survival probability γ of diapausing eggs is dependent on the minimum winter temperature, T_DJF,min_. The survival probability is applied when eggs are activated in spring. Remaining diapausing eggs that have not hatched until August are removed.

**Table 1:** Parameter definitions and values. References for point estimates are given below, detailed derivation and references of all other parameters are shown in [28]. Environmental drivers are temperature, T, rainfall, R, photoperiod, P, latitude, L, day of year, DOY, and human population density, H.

	Parameter	Value/Formula
CTT_S_	Critical temperature in spring (°C)	11.0 *
CPP_S_	Critical photoperiod in spring (hours)	11.25 *
*φ*(T, P)	Spring hatching rate (1/day)	0 if T_7_ < CTT_S_ or P < CPP_S_ 0.1 if T_7_ ≥ CTT_S_ and P ≥ CPP_S_
CPP_A_(L)	Critical photoperiod in autumn (hours)	10.058 + 0.08965 L
ω(P)	Fraction of eggs going into diapause	0 if DOY < 183 or P > CPP_A_ 0.5 if DOY ≥ 183 and P ≤ CPP_S_
τ_E_	Normal egg development time (days)	7.1
τ_J_(T)	Juvenile development time (days)	83.85 − 4.89T + 0.08T^2^
τ_I_(T)	First pre-blood meal time (days)	50.1 − 3.574T + 0.069T^2^
μ_E_(T)	Normal egg mortality rate (1/day)	−ln(0.955 exp(−0.5 ((T-18.8)/21.53)^6^))
μ_J_(T)	Juvenile mortality rate (1/day)	−ln(0.977 exp(−0.5 ((T−21.8)/16.6)^6^))
μ_A_(T_mean_)	Adult mortality rate (1/day)	− ln(0.677 exp(−0.5 ((T_mean_-20.9)/13.2)^6^) T_mean_^0.1^)
γ(T_DJF,min_)	Survival of diapausing eggs (1/winter)	0.93 exp(−0.5 (T_DJF,min_-11.68)/15.67)^6^)
β(T)	Egg laying rate (1/day)	33.2 exp(−0.5 ((T−70.3)/14.1)^2^) (38.8 − T)^1.5^ if T ≤ 38.8 0 if T > 38.8
λ	Capacity parameter (larvae days/hectare)	40^4^ ^†^

* [74]

^†^ Estimated so that the maximum vector-to-host ratio in Guangzhou was about 100 adult females per human, compared to 94–314 bites per person per day in nearby Macau [75].

The carrying capacity K depends on rainfall R and human population density H, and is given by

$K(R,H)=\lambda \frac{1-\alpha_{evap}}{1-\alpha_{evap}^{t}}\sum_{x=1}^{t}\alpha_{evap}^{\left(t-x\right)}(\alpha_{rain}R(x)+\alpha_{dens}H(x))$ with scaling factors α_evap_, α_rain_, and α_dens_ as defined in [28,29].

Note that we do not follow the approach for delay differential equation models by [20] in which the time delay itself is modelled by a differential equation. Instead, we calculate all time delays before we run the simulations, see S1.2 in S1 Supplementary Material.

### Disease dynamics in the vector

**Fig 2:**
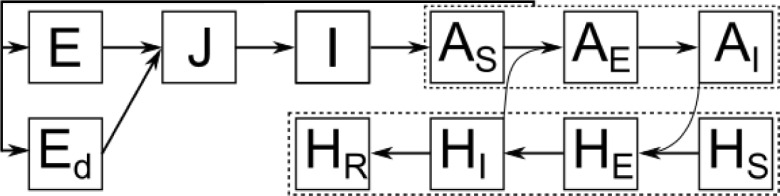
The mosquito life cycle with the mature female class split into susceptible, exposed, and infectious females, A_S_, A_E_ and A_I_, respectively. When a susceptible mosquito bites an infected human host, it gets infected (exposed) with a certain probability and changes to the infectious class after a temperature-dependent extrinsic incubation period. Human hosts change from susceptible, H_S_, over exposed, H_E_, to infectious, H_I_, and finally recover, H_R_. Parameter symbols and mortality arrows are not included for readability.

An introduction of dengue virus (DENV) into the model leads to two more vector classes. We now distinguish between susceptible mosquito females, A_S_, exposed females that are infected but cannot transmit the virus yet, A_E_, and infectious females, A_I_, see Fig 2.

When a host seeking female bites an infected human with the biting rate α, it will become exposed with a certain probability b_HV_. With H_I_/N_h_ as the fraction of infected humans in the local population, this leads to a total infection rate of αb_HV_H_I_/N_h_ for susceptible mosquitoes, A_S_. Depending on the temperature-dependent extrinsic incubation period (EIP), exposed females will finally change to the infectious state during which they can infect human hosts. All susceptible, exposed and infected females die with the normal adult mortality, μ_A_ and continue to lay eggs with fecundity rate β. Eq 5) for mature females becomes

$$\frac{d}{dt}A_{S}\left(t\right)=M_{I}\left(t\right)-\alpha b_{HV}\frac{H_{I}\left(t\right)}{N_{h}\left(t\right)}A_{S}\left(t\right)-\mu_{A}A_{S}\left(t\right)$$ (6)

$$\frac{d}{dt}A_{E}\left(t\right)=\alpha b_{HV}\frac{H_{I}\left(t\right)}{N_{h}\left(t\right)}A_{S}\left(t\right)-\alpha b_{HV}\frac{H_{I}\left(t-EIP\right)}{N_{h}\left(t-EIP\right)}A_{S}\left(t-EIP\right)exp\left(-\int_{t-EIP}^{t}\mu_{A}\left(\sigma \right)d\sigma \right)-\mu_{A}A_{E}\left(t\right)$$ (7)

$$\frac{d}{dt}A_{I}\left(t\right)=\alpha b_{HV}\frac{H_{I}\left(t-EIP\right)}{N_{h}\left(t-EIP\right)}A_{S}\left(t-EIP\right)exp\left(-\int_{t-EIP}^{t}\mu_{A}\left(\sigma \right)d\sigma \right)-\mu_{A}A_{I}\left(t\right),$$ (8)

such that the three adult female classes combined result in the total number of egg-laying females: A_S_ + A_E_ + A_I_ = A.

Parameter values are given in Table 2. Note that the incidence rate αb_HV_ H_I_/N_h_ A_S_ uses non-delay terms in Eq (6) but delay terms in Eq (8). This issue is explained in more detail in S1.3 in S1 Supplementary Material.

**Table 2:** Additional parameter definitions and values used for disease dynamics with references at the bottom.

	Parameter	Value/Formula
α(T)	Biting rate (1/days)	0.5 (0.0043 T + 0.0943) *
b_VH_	Infection probability from vector to host	0.5 ^†^
b_HV_	Infection probability from host to vector	0.31 ^†^
EIP(T)	Extrinsic incubation period (days)	1.03 (4 + exp(5.15–0.123T)) ^‡^
δ	Max. number of people infected by single mosquito (individuals / hectare)	4.5
IIP = ^1^/ν_H_	Intrinsic incubation period (days)	5^§^
r	Recovery rate of humans (1/days)	⅕^§^

* Daily biting rates for *Ae*. *aegypti* vary between 0.1 and 0.2 blood meals per day [76,77], halved for *Ae*. *albopictus* based on observed feeding intervals [78].

^†^ Estimates by [79,80].

‡ Estimate for *Ae*. *aegypti* by [76] with scaling factor for *Ae*. *albopictus* by [81].

¶ Estimate for Guangzhou 2014.

§ Estimates by [82,30].

This approach neglects the possibility of vertical dengue virus transmission, i.e. the potential transmission of the virus from adult females to their eggs and thus to the next generation of adults. In consequence, there is no overwintering of infected eggs that could start a new outbreak in the following year and new dengue cases would have to be imported to initiate dengue transmission.

### Disease dynamics in the human host

We model disease dynamics in the human host with ordinary differential equations (ODEs) as the incubation period in the human host is relatively short and temperature-independent, and ODEs are computationally less expensive to solve.

Human hosts are described by four classes, susceptible H_S_, exposed H_E_, infected H_I_, and recovered H_R_. When a susceptible human host, H_S_, gets bitten by an infected mosquito, it changes to the exposed state, H_E_, with a probability of b_VH_. It remains in the exposed state for the intrinsic incubation period (IIP) and then changes to the infected state, H_I_. Infected individuals then recover with a rate of r and become immune, H_R_. We assume that we only have a single dengue virus serotype circulating in the population. As we look at very small numbers of infections for only short periods, we neglect birth and natural death rates for human hosts as well as (cross-)immunity to serotypes. We also neglect dengue induced mortality as it is usually very low (below 1% [30]) and thus has only a marginal effect on disease dynamics. Equations for infection dynamics in the human host are then given by:

$$\frac{d}{dt}H_{S}\left(t\right)=-\alpha b_{VH}A_{I}\left(t\right)\frac{\delta }{N_{h}\left(t\right)}H_{S}\left(t\right)$$ (9)

$$\frac{d}{dt}H_{E}\left(t\right)=\alpha b_{VH}A_{I}\left(t\right)\frac{\delta }{N_{h}\left(t\right)}H_{S}\left(t\right)-\nu_{H}H_{E}\left(t\right)$$ (10)

$$\frac{d}{dt}H_{I}\left(t\right)=\nu_{H}H_{E}\left(t\right)-r\;H_{I}\left(t\right)$$ (11)

$$\frac{d}{dt}H_{R}\left(t\right)=r\;H_{I}\left(t\right)$$ (12)

Note that we divide the infection probability term for the vector in Eq (6), αb_HV_H_I_/N_h_A_S_, by the total number of humans, N_h_ (frequency-dependent transmission) [31], while we do not divide the infection probability term for the human, αb_VH_A_I_δ/N_h_H_S_, by the total number of mature mosquitoes, A (density-dependent transmission), in Eq (9). Dividing the infection probability of the vector by the total number of human hosts accounts for a diluting effect, so that an infected person in a big city is less likely to be bitten than an infected person in a rural area if we assume the same mosquito abundance. This diluting effect does not apply to mosquitoes equally. We assume that the number of newly infected persons depends on the total number of infected mosquitoes in an area, rather than on the frequency of infected females in the mosquito population. We do, however, introduce the scaling factor δ/N_h_; a single mosquito will thus only infect H_x_ = αb_VH_δ humans per day.

To investigate the influence of each parameter on the model output we perform the elementary effects test (EET), see S1.4 in S1 Supplementary Material for details.

### Climate and population data

We use weather station data from the Chinese Meteorological Data Service Center (CMCD). The network comprises more than 800 stations with daily temperature and rainfall data available from 2006 to 2016. Missing rainfall data for weather stations in the CMCD data set are imputed with the nearest neighbour technique [32]. Human population density is based on the GPWv4 data set for 2015 [33], using the values of the grid cells in which weather stations are located. Data points are linearly interpolated on a grid of approx. 2 km by 2.75 km for filled contour plots.

For the four cities, we use data point #133 for Beijing (40.23°N, 116.52°E), #86 for Jinan (36.36°N, 117.00°E), #362 for Shanghai (31.24°N, 121.27°E) and #760 for Guangzhou (23.13°N, 113.29°E) from the CMCD data set. Note that the nearest weather station for Beijing is located about 39 km outside the city centre in a sparsely populated area. We thus use population densities of 13, 9.5, 38, and 21.06 humans per hectare for Beijing, Jinan, Shanghai, and Guangzhou respectively [33–37]. These densities are higher than the values of their nearest weather station grid cell but more accurate. However, this leads to discrepancies between regional and city-level results.

### Mosquito and dengue data

We use China wide presence/absence data for *Ae*. *albopictus* for 2015 (S1 Data) to validate our vector suitability predictions. We run the model with the climate data set for the period 2006 to 2016 and calculate individual suitability indices. The suitability index is defined by the number of eggs that are produced at the end of a year, after placing a single (diapausing) egg at the beginning of that year into an uncolonised location [28]. The ratio by which the number of eggs has increased (suitable) or decreased (unsuitable) defines the suitability index of that year, and we took the median over the simulated 10 years. We then use a nearest neighbour algorithm to match presence/absence locations with weather station locations. We check performance and optimal threshold of the suitability index with a receiver operating characteristic (ROC) analysis.

The Breteau index (BI) measures the number of water containers or bodies infested with mosquito larvae per 100 estates or houses inspected. Monthly BI data of *Ae*. *albopictus* was available for Guangzhou for 2006–2015 to validate population dynamics over longer time periods (S2 Data). BI data was also available for 21 out of 34 provinces, autonomous regions or municipalities for 2015 to spatially validate population dynamics of the vector (S3 Data). To estimate a BI from mosquito data, we assume that clutches of eggs are randomly (Poisson) distributed to available containers. The probability that a container contains one or more juveniles, multiplied by the number of containers available, gives the BI estimate:

$$BI\left(t\right)=b(1-e^{-aJ\left(t\right)})$$ (13)

J(t) is the total number of juvenile larvae and pupae in the area at time t, *a* is a factor to relate total juvenile numbers to the mean number per container and *b* is the mean number of water containers per 100 households. Here, we use *b* = 140 [38] and *a* = 0.00054. The factor *a* is calculated as the inverse of the average number of juveniles per container (4.45L/container * 42.3 juv/L, [39]), multiplied by the average number of containers per area (1.4 container/household * 21.06 people/hectare / 3 people/household [40]). BI values above 4 can be a good indicator for dengue transmission risk [41,42], however BI often does not correlate with dengue incidence and outbreaks can happen with BI < 4 [43] or lagged by up to three months [44].

Dengue data was available for 2014 to validate the modelled infection dynamics. The 2014 dengue outbreak took place mostly in Guangdong province, with its capital Guangzhou as the epicentre, but also subsequently spread to other provinces. To analyse weekly case numbers for Guangzhou only, we included all cases that happened within a 72 km radius around the Guangzhou city centre (23.09°N, 113.17°E). This radius was chosen to also account for the large number of cases reported in the directly adjacent urban areas of Guangzhou, see Fig D in S1 Supplementary Material for details.

Mosquito and dengue incidence data sets were recorded and provided by the National Institute for Communicable Disease Control and Prevention (ICDC), China CDC. Vector and disease occurrence locations were given at prefecture (300+) or county (2500+) level, and thus had to be pre-processed to allocate mosquito occurrences or disease cases to locations in coordinate format. The geo-location website http://www.geonames.org/ was used to retrieve latitude and longitude coordinates for centres of administrative regions. To validate the results, the matching algorithm was repeated with a random sample using another database at http://maps.cga.harvard.edu/ to double check the accuracy coordinates.

To calculate the season length of potential dengue transmission, it is assumed that every single emerging adult female meets a dengue-infected human for its first blood meal, from the first female in the season to the very last one. Simulations show that Guangzhou experienced infectious mosquito densities of at least one infected mosquito per hectare during the outbreak 2014, roughly from early May to mid-December. The maximal length of the dengue transmission season is thus defined as the period with at least one infected mosquito per hectare in this setting.

## Results

Analytical and numerical solutions show that the vector population can persist between 16.5 and 32.5°C, with an optimal temperature of 27°C, see S1.6 in S1 Supplementary Material. Depending on the temperature, the model predicts that the larval and pupal population is about 4 (around the optimal temperature) to 30 times (towards the lower and upper suitable ranges) larger than the adult population.

### Mainland China’s suitability for *Ae*. *albopictus*

**Fig 3:**
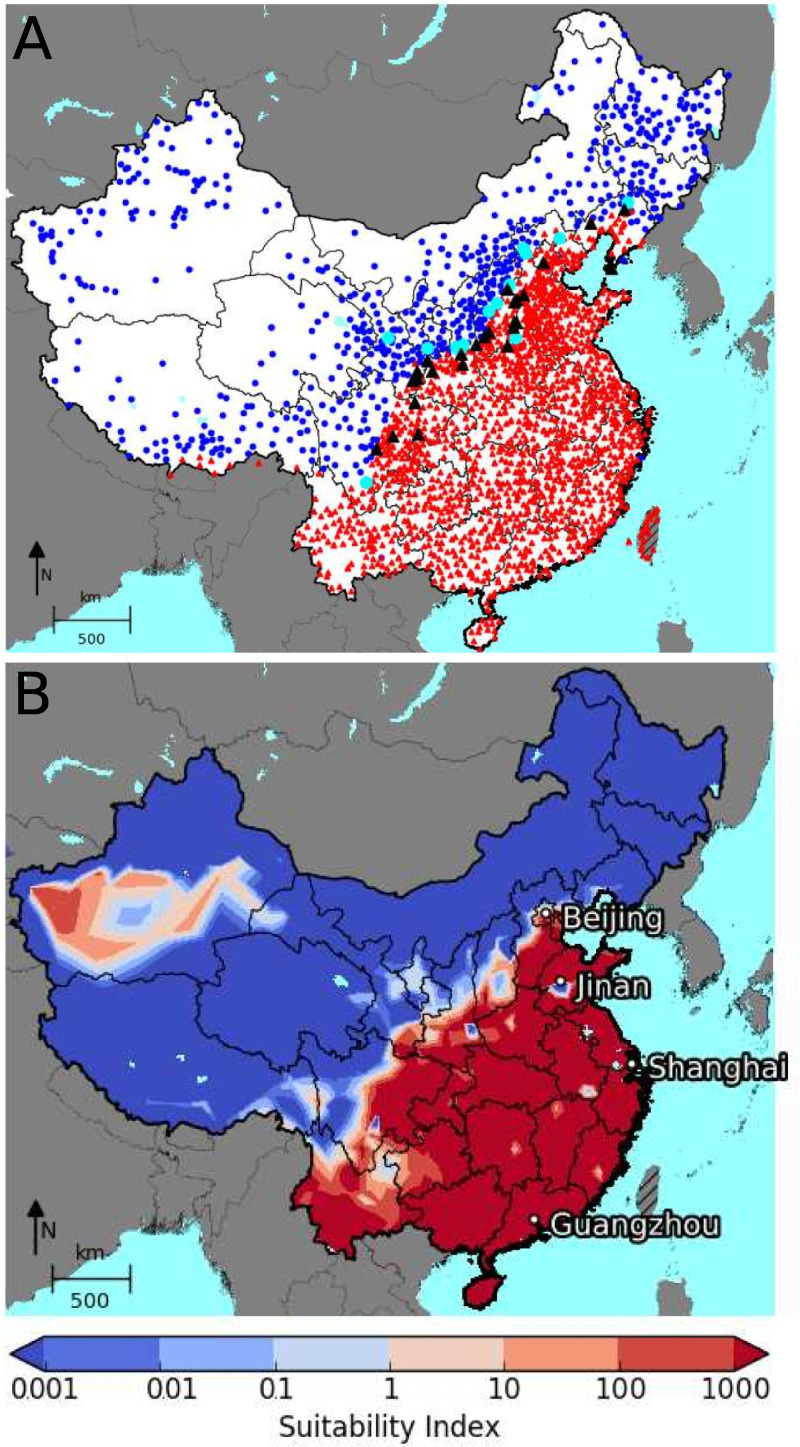
Mainland China’s suitability for *Ae*. *albopictus*. Top: Mosquito presence in 2007 (black triangles) and 2015 (red triangles) and absence for 2007 (cyan dots) and 2015 (blue dots). Bottom: Modelled suitability index (E_0_) for the period 2006 to 2016. Values above 1 indicate suitable regions, below unsuitable regions. The CMCD climate data set was used to drive the model. Maps were created with Python package Basemap [83].

Fig 3 shows *Ae*. *albopictus* presence/absence data derived from [12] and collected by China CDC, in comparison with the modelled suitability index. The modelled suitability is highly consistent with presence/absence data: 90.4% of data points are correctly categorised as present/absent, the sensitivity is 92%, the specificity is 87% and Cohen’s κ = 0.77. The central plain and the southern hills are highly suitable for *Ae*. *albopictus*, while the Tibetan plateau and northern China appear unsuitable. However, the model misses some suitable locations in the Himalayas close to the border with Bhutan, as well as in areas east of Beijing, as far as the border with North Korea. Both regions experience winters with freezing temperatures below -15°C. This makes overwintering unlikely by model assumptions, but microclimate conditions could help *Ae*. *albopictus* to survive even here, e.g. in some suitable mountain valleys, and habitations in warmer cities.

On the other hand, the model falsely predicts suitable areas in the Taklamakan desert in Xinjiang Uygur Autonomous Region in western China. While the temperatures would be suitable here, the scarce rainfall probably prohibits mosquito activity. There are, however, oases and the cities Kashgar and Yarkant with more than 100,000 inhabitants, rivers and relatively mild diurnal temperature ranges on the south-western edge of the desert that could enable mosquito larvae development in artificial breeding sites.

**Fig 4:**
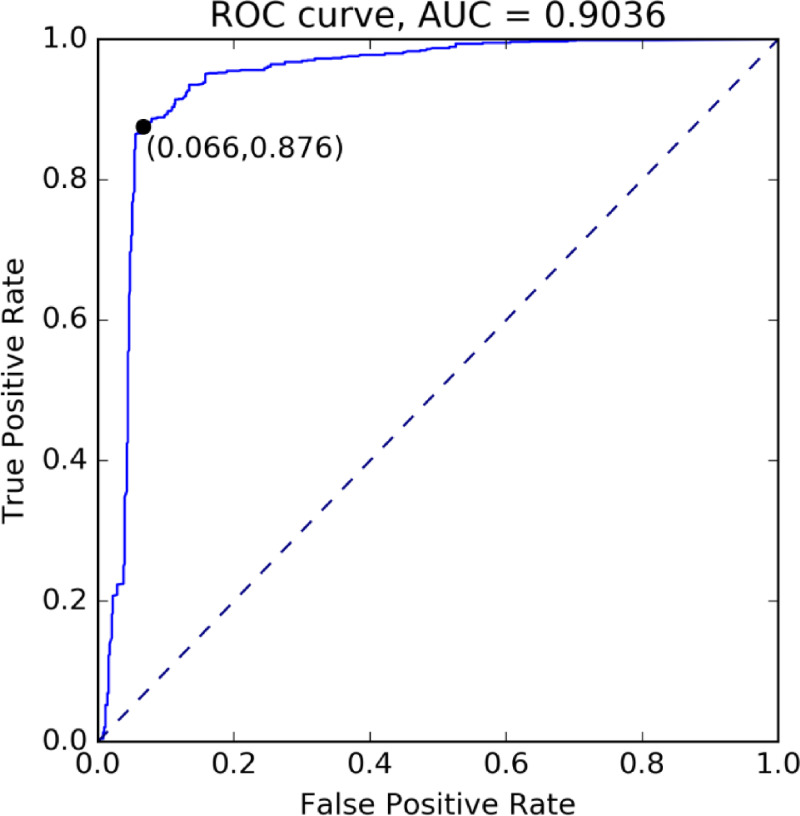
Receiver operating characteristic curve. The black dot represents Youden’s index, the best compromise between specificity (1-False Positive Rate) and sensitivity (True Positive Rate). The area under the curve (AUC) gives the probability of a region with mosquito occurrence getting a higher suitability index than one without.

The ROC analysis found that the rate of correctly predicted suitability could be slightly improved when we defined areas as suitable where one egg leads to about 25 eggs after 365 days, see Fig 4. With this adapted suitability index, the Taklamakan desert would show up as unsuitable while the south and east remain suitable, see S1.7 in S1 Supplementary Material for further details.

### Mosquito dynamics

**Fig 5:**
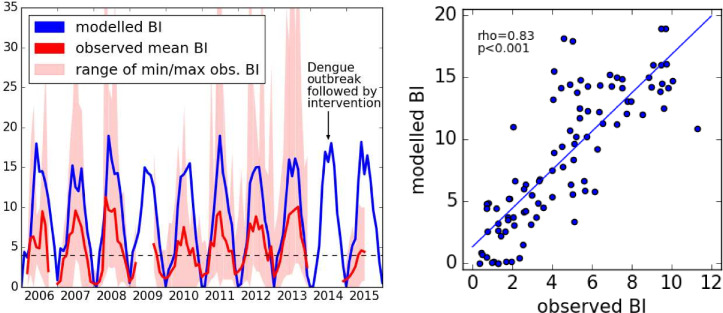
Monthly Breteau indices (BIs) in Guangzhou. Left: The modelled BI in Guangzhou compared to the observed value. BI values above 4 (dashed black line) indicates risk of dengue transmission. Note that BI data for 2009 and 2014 were incomplete. Right: Scatter plot of simulated vs. observed BI values.

In the next step, we checked whether the DDE model could replicate temporal mosquito dynamics and compared model results with Breteau index (BI) data for Guangzhou from 2006 to 2015. Monthly Breteau indices in Guangzhou were measured at 12 different locations. Two locations (Nansha and Panyu) were situated at the river delta and near a wetland park and showed very high BI values of up to 60 positive containers per 100 houses. Monthly mean model outputs fit the observed BI values well (Spearman’s ρ = 0.83, p<0.001). BI values peak in summer and are low in winter and correctly indicate years with higher (2008) and lower (2010) numbers than average, see Fig 5. In 2015, intensive mosquito control measures were undertaken following the 2014 outbreak, consequently fewer mosquitoes were observed. Our model, that does not consider control measures, overestimated BI in 2015.

Observed BI values for Guangzhou in winter are often well above zero. This mosquito activity during winter is not captured by the model, which assumes very low survival rates below 0°C. We also compared model outputs to monthly BI data at province level for 2015. While our model predicts the highest mosquito numbers in southern China, recorded BI numbers in 2015 were actually highest in some northern provinces, see S1.8 in S1 Supplementary Material.

### Dengue outbreak in Guangzhou 2014

We now introduce dengue transmission in our DDE model framework and simulate the 2014 dengue outbreak in Guangzhou.

**Fig 6:**
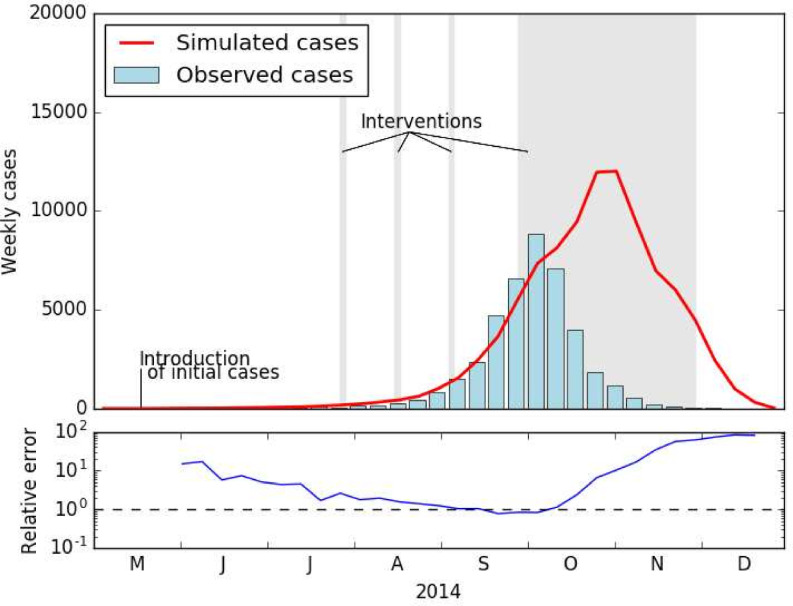
Dengue cases in Guangzhou. Top: Observed number of weekly human dengue cases from May to December during the 2014 dengue outbreak (blue bars) and simulated weekly cases (red line). Grey shades indicate officially ordered interventions to control the mosquito population [13]. Bottom: Dividing simulated by observed incidence cases gives the relative error.

Fig 6 shows the number of weekly cases during the 2014 dengue outbreak in the Guangzhou area. In response to the ongoing outbreak, mosquito control measures or interventions (container emptying and fumigation with chemical treatments) were conducted. These vector control measures were applied sporadically in the summer months and then continuously throughout October and November [13].

Simulated weekly cases are very realistic during the first half of the outbreak. Following the introduction of 691 infected cases in mid-May [45], the number of weekly cases slowly increases throughout June, July and August before seeing a steep increase in September. While observed cases decreased from October onwards, possibly due to intervention efforts, simulated case numbers continued to increase for another four weeks until unfavourable climate conditions very likely limited the outbreak in December.

**Fig 7:**
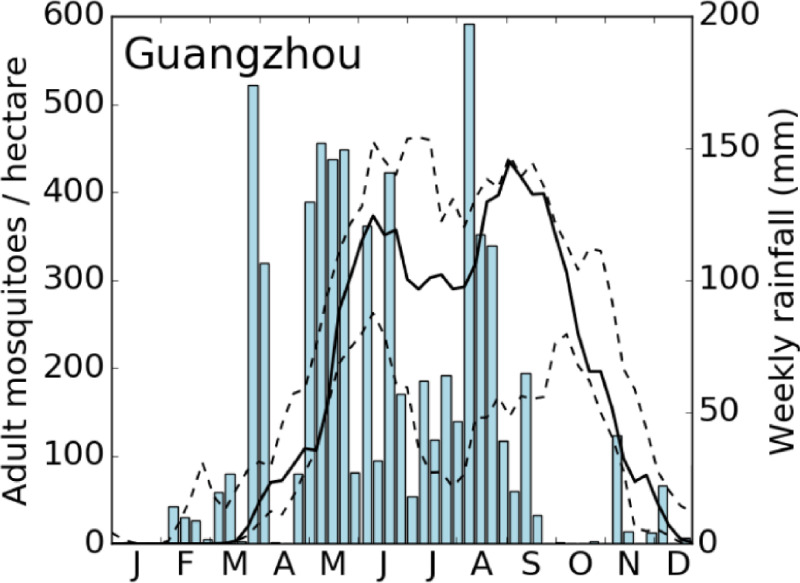
Mosquito density in Guangzhou 2014. Solid line: Modelled adult females (uninfected and infected) during the 2014 dengue outbreak. Dashed lines: Simulated maximum and minimum adult female density during the period 2006–2016. Blue bars: Weekly rainfall. Heavy rainfall in May might have led to the steep increase in observed mosquito numbers in late May/June.

An introduction of dengue cases in mid-May was immediately followed by a rapid increase in mosquito abundance (Fig 7). While May temperatures in Guangzhou are generally very suitable for *Ae*. *albopictus*, there was above-average rainfall in May 2014 that likely created additional breeding sites. Modelled mosquito density for 2014 was higher than the modelled ten-year average, particularly in June, August and September which might have added to the outbreak.

### Simulated Length of the dengue transmission season

**Fig 8:**
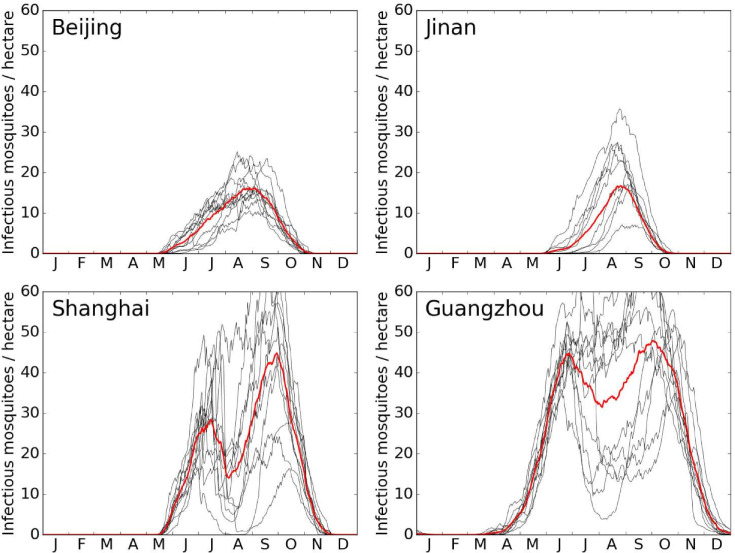
Maximum density of infected mosquitoes. Black lines indicate simulations for individual years 2006–2016, the red lines indicate means. Note that these high infectious mosquito densities were only reached if every bite occurred on an infected human. During the 2014 outbreak, infectious mosquito numbers were about 0.0002 infectious females per hectare in our simulation.

The potential length of the dengue transmission season for China is analysed. Fig 8 shows the maximum number of infected mosquitoes for four key Chinese cities: Beijing, Jinan, Shanghai and Guangzhou. Beijing has a relative short mosquito season, with potentially infectious mosquitoes from June/July to October. Jinan, a city with 8.5 million inhabitants, about 400 km south of Beijing, shows similar mosquito densities with higher peaks in mid-summer in individual years. Shanghai and Guangzhou show a different pattern. Here, numbers already start to increase in April/May and decline to near-zero only in December. Moreover, the curves show a strong dip during the hot summer months. The maximal length of the dengue seasons would be about 3 months for Beijing and Jinan, about 6 months for Shanghai and could last up to 8 months for Guangzhou. We also repeated these simulations using the equivalent ODE model that gave much higher numbers and longer dengue seasons, especially for the more temperate regions, see S1.9 in S1 Supplementary Material.

**Fig 9:**
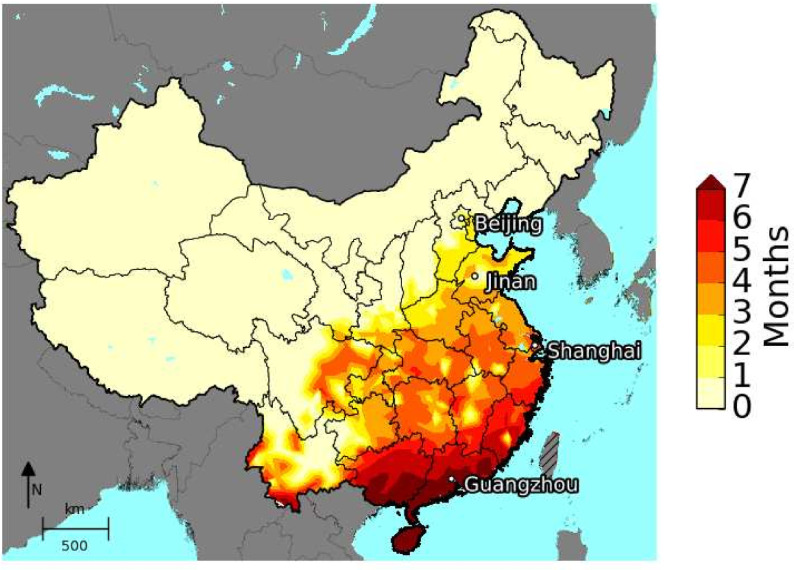
Simulated length of the dengue transmission season (months). The depicted median was calculated over the 2006–2016 period. Beijing, Jinan, Shanghai and Guangzhou are indicated by black dots. The map was created using the Basemap package in Python package [82].

Analysing the maximal length of the dengue transmission season for mainland China shows that dengue transmission is theoretically possible nearly everywhere *Ae*. *albopictus* is present, see Fig 9 and Fig 3. The south coast of China including Hainan, Guangxi, Guangdong, and Fujian, shows the longest transmission season of seven months and more. The further north, the shorter the simulated length of the dengue transmission season. Areas near but outside the urban areas of Beijing only have about six weeks where dengue might be effectively transmitted. Cities with higher human population densities such as Beijing and Guangzhou have an even longer transmission period when simulated individually (Fig 8). This is since the climate data is often derived from weather stations outside bigger cities where the population density is lower, which leads to fewer artificial breeding sites simulated by the model.

## Discussion

Though vector population and disease dynamics are difficult to predict from environmental factors, we realistically modelled the temperature-dependence of *Ae*. *albopictus*, its seasonal and local abundance and its role in dengue transmission in mainland China with a novel stage-structured DDE model. The model gives basic species’ characteristics, such as temperature range and temperature optimum, that are in line with statistical analyses on this species’ temperature niche [46,47].

The simulated length of the dengue transmission season for mainland China corresponds very well with observed transmission between 2002 and 2008 for southern China. With a few exceptions, all cases happened within the seven months of May/June to December [8]. The areas with the longest transmission periods are also in line with the identified areas at risk of dengue for the 2020s by [48]. These areas are situated on the south coast of China because here, the maximum number of infectious mosquitoes is greatest and potential outbreaks have more time to build up. Further north, the model still predicts dengue transmission as far north as Beijing (one to two months). Shandong province, close to Beijing with up to three months of potential transmission, recently experienced a minor dengue outbreak in 2017 with about 200 cases [49] and in 2019 with about 51 cases. The simulated density of infectious mosquitoes is even higher for individual cities and the potential transmission period is longer than in the surroundings, due to the model’s assumption that this *Aedes* species thrives better in urban areas with higher human population densities.

Looking at the 2014 dengue outbreak in Guangzhou, model simulations suggest that the outbreak could have reached much higher numbers if it was not for the officially ordered control interventions. This finding is in accordance with other modelling studies [13,17]. It should be noted though that up to 75% of dengue cases are asymptomatic [50] and that actual case numbers during the outbreak were much higher. The omission of asymptomatic infections in our model framework might lead to an underestimation of the number of cases, especially at the start of the outbreak. While temperatures were not exceptionally suitable during the outbreak for this region [16], the early introduction of initial, imported dengue cases in May led to a long build-up of case numbers that played a key role in the final outbreak size [13]. Our results corroborate that this build-up of sufficient infectious mosquito numbers was only possible in southern regions of China.

While the model had a good performance in simulating mosquito presence and absence, between-year mosquito abundance and the length of the dengue transmission season, it did not fully capture mosquito activity during the winter months, possibly due to two reasons: larvae could endure low outside temperatures for longer time periods than laboratory studies that were used to drive our model parameters [51] suggest. Field studies in southern as well as in northern provinces would be helpful to better tailor mosquito survival parameters in the model to winter temperatures, especially as different strains of *Ae*. *albopictus* might show different climate responses or indoor/outdoor resting behaviour. It is also possible that larvae inhabit warmer households indoors in winter and thus survive longer due to microclimatic conditions which are not considered by the model. However, houses in southern China often lack heating systems, consequently this factor might play less of a role in Guangzhou compared to northern provinces of China or Europe. Another study has found lower, but still positive mosquito indices during Guangzhou winter months [52].

The model also struggled to reproduce observed BI values for 2015, both for the local data in Guangzhou, as well as for regional data in Chinese provinces. An explanation for this discrepancy might be that significant mosquito control interventions were undertaken in 2014 and 2015 [13], especially in the southern provinces which skewed observed BI values. In addition, the modelled regional BIs represent means of random samples of locations, which might not be the case for recorded province-level BIs, for which locations with known established mosquito populations might have been chosen.

*Ae*. *albopictus* was the main vector responsible for this outbreak. Li et al. showed that this mosquito species is getting harder to control due to increased insecticide resistance in recent years [53]. In contrast to Europe where this mosquito species might spread farther north in the future [28], model results suggest that *Ae*. *albopictus* has already filled its full potential ecological niche in mainland China, and this finding is consistent with observations for 2007 [12]. Nevertheless, it might spread farther north in the future as warming temperatures render regions in the north and at higher altitudes more suitable [54].

The yellow fever mosquito, *Ae*. *aegypti*, which is more competent to transmit dengue virus than *Ae*. *albopictus*, is currently only present on Hainan island, Leizhou peninsula in Guangdong and bordering counties in Yunnan province [55]. However, urbanisation and warming temperatures might also favour *Ae*. *aegypti* to spread farther north and east [56] and the future could see both *Aedes* species overlapping in Guangzhou and Guangdong provinces [57]. This will also lead to further spread of dengue outbreaks [58] and probably also affect other arboviruses circulating in mainland China [2], including the Zika virus [59] and Batai virus [60].

Apart from the presence of the vector, climate is one of the most important factors for mosquito-borne disease transmission in mainland China [61]. While we modelled the climate dependencies of mosquito and virus very carefully, we had to neglect other possible drivers of disease emergence. These included socioeconomic factors such as the regional gross domestic product (GDP) [62], differences in insecticide usage [63], but also the possibility for *Ae*. *albopictus* to pass on the dengue virus from adult female to egg, the so-called vertical transmission [64]. Vertical transmission of dengue virus has been observed in many south-east Asian countries [65], but it is still debatable to what extent this mechanism actually contributes to disease outbreaks [66]. In their study, Sun et al. concluded that vertical dengue virus transmission was unlikely to have impacted the 2014 outbreak [9].

However, recent literature suggests that some dengue serotypes might have circulated in southern China for years [67] and that the dengue strain responsible for the 2014 outbreak has been circulating since [68]. It is an interesting question whether this is still caused by the aftermath of the 2014 outbreak and will vanish in the next few years or whether dengue has to be considered endemic in China again. Future modelling studies could include different dengue serotypes [69] and thus more realistically predict severe dengue cases with potential cross reactions. Finally, future studies could also introduce stochasticity in mosquito parameters or regional habitat qualities to further assess model uncertainty [70,71]. It is possible that the latter could explain some of the discrepancies observed in our regional BI predictions.

While this model indicates the constant risk of dengue transmission, it can also indicate where and when *Ae*. *albopictus* populations build up to large numbers such as during the summer months in Guangzhou in 2014. Our abundance model could then be combined with the statistical model developed by Sang et al. in order to build an early warning system to anticipate dengue hotspots in real time [72]. These spatial hotspots could be suppressed by targeted vector control measures [73] before an introduction of the virus leads to thousands of dengue cases again.

In summary, we show that DDE models are a valuable tool in explaining and predicting vector-borne disease outbreaks, here capturing many aspects of the recent epidemiology of dengue virus transmission dynamics in mainland China and the distribution of one of its key vectors, *Ae. albopictus*.

## Supporting information

S1 Data China-wide county-level presence/absence data for *Ae*. *albopictus* from 2015. (CSV)

S2 Data Monthly Breteau Index data for *Ae*. *albopictus* for 12 locations in Guangzhou, Guangdong province from 2008–2015. (CSV)

S3 Data Monthly Breteau Index data for *Ae*. *albopictus* for 21 Chinese provinces, autonomous regions or municipalities from 2015. (CSV)

S1 Supplementary Material Supporting information S1.1 - S1.9. (PDF)
