# Supplementary material for: Assessing the suitability for Aedes albopictus and dengue transmission risk in China with a delay differential equation model
Source: PLoS Negl Trop Dis. 2021 Mar 26;15(3):e0009153. doi: 10.1371/journal.pntd.0009153 (PMC7996998; doi:10.1371/journal.pntd.0009153)
Supplement: S1 Supplementary Material — (PDF) [file pntd.0009153.s004.pdf]

S1 Supplementary Material

Assessing the suitability for *Aedes albopictus* and dengue transmission risk in China with a delay differential equation model

Metelmann, Liu, Lu, Caminade, Liu, Cao, Medlock, Baylis, Morse, Liu

Contents

S1 Supplementary Material **1**
S1.1 Deriving DDEs from ODEs . . . 2
S1.2 Time delay calculation . . . 4
S1.3 ODE vs. DDE dynamics . . . 5
S1.4 Sensitivity Analysis . . . 8
S1.5 Pearl River Delta . . . 9
S1.6 Equilibria . . . 10
S1.7 Updated suitability . . . 14
S1.8 China-wide BI data 2015 . . . 15
S1.9 DDE vs. ODE transmission risk . . . 16

List of Figures

A DDE vs. ODE - Early disease onset . . . 6
B DDE vs. ODE - Effect of EIP on models . . . 7
C Elementary effects test . . . 8
D Pearl River Delta . . . 9
E Temperature range . . . 13
F Suitability for *Ae. albopictus* with new threshold . . . 14
G Monthly BI values . . . 15
H DDE vs. ODE - mosquito model comparison . . . 16

S1.1 Deriving DDEs from ODEs

The terms contributing to the rate of change for each class can be classified into three categories: recruitment, maturation, and mortality. The transformation is demonstrated with the help of class E, eggs.

We assume there are E_0 eggs at $t = t_0$ and, for simplicity, that no eggs are laid after $t = t_0$. Eggs experience a temperature-dependent mortality of $\mu(T(t)) = \mu(t)$:

$$\begin{aligned} E(t_0) &= E_0 \\ \tfrac{d}{dt}E(t) &= -\mu(t)\, E(t). \end{aligned}$$

This equation can easily be integrated to

$$E(t) = E_0\, e^{-\int_{t_0}^{t} \mu(\sigma)\, d\sigma}$$

and defining $t^\star = t - t_0$ gives

$$E(t) = E(t - t^\star)\, e^{-\int_{t-t^\star}^{t} \mu(\sigma)\, d\sigma}.$$

After an egg development period of τ_E, all eggs mature to the juvenile stage, thus at $t = \tau_E$:

$$\begin{aligned} \tfrac{d}{dt}E(t)|_{t=\tau_E} &= -\delta_{\tau_E} E(t) \\ &= -\delta_{\tau_E} E(t - \tau_E)\, e^{-\int_{t-\tau_E}^{t} \mu(\sigma)\, d\sigma} \\ &= -M_E(t) \end{aligned}$$

with $\delta_{\tau_E} = 1/\Delta t$ at τ_E and 0 otherwise. Finally, we include egg recruitment. Class E is recruiting from mature females A with a rate of $\beta\,(1-\omega)$ (egg laying rate times a factor for diapausing eggs).

$$\begin{aligned} \tfrac{d}{dt}E(t) &= \beta\,(1-\omega)\, A(t) \\ &= R_E(t) \end{aligned}$$

and

$$E(t_0) = \beta\,(1-\omega)\, A(t_0).$$

Combining recruitment, maturation, and mortality gives

$$\begin{aligned} \tfrac{d}{dt}E(t) &= R_E(t) - M_E(t) - \mu(t)\, E(t) \\ &= \beta\,(1-\omega)\, A(t) - E(t-\tau_E)\, e^{-\int_{t-\tau_E}^{t} \mu(\sigma)\, d\sigma} - \mu(t)\, E(t) \\ &= \beta\,(1-\omega)\, A(t) - (\beta\,(1-\omega)\, A(t-\tau_E))\, e^{-\int_{t-\tau_E}^{t} \mu(\sigma)\, d\sigma} - \mu(t)\, E(t). \end{aligned}$$

The same applies to immature female development with a development period of τ_I, and transition of exposed to infectious mosquitoes after the EIP.

Due to larval density dependence and the additional mortality term, juvenile mortality does not follow an

exponential decay. Integration of

$$\tfrac{d}{dt} J(t) = -\mu(t)\, J(t) - \frac{J(t)^2}{K(t)}$$

is achieved by substitution of $x(t) := J(t)\, \exp(\int_0^t \mu(\sigma)\, d\sigma)$. Then

$$x_0 = x(0) = J(0) = J_0$$

and

$$\begin{aligned}
\tfrac{d}{dt} x(t) &= \tfrac{d}{dt}\left(J(t)\, \exp\left(\int_0^t \mu(\sigma)\, d\sigma \right) \right) \\
&= \left(\tfrac{d}{dt} J(t) + \mu(t)\, J(t) \right) \exp\left(\int_0^t \mu(\sigma)\, d\sigma \right) \\
&= \left(-\mu(t)\, J(t) - \frac{J(t)^2}{K(t)} + \mu(t)\, J(t) \right) \exp\left(\int_0^t \mu(\sigma)\, d\sigma \right) \\
&= -\frac{J(t)^2}{K(t)} \exp\left(\int_0^t \mu(\sigma)\, d\sigma \right) \\
&= -x(t)^2 \frac{1}{K(t)} \exp\left(- \int_0^t \mu(\sigma)\, d\sigma \right).
\end{aligned}$$

Integration yields

$$x(t) = \frac{1}{\int_0^t \frac{1}{K(\xi)} \exp(-\int_0^\xi \mu(\sigma)\, d\sigma) d\xi + c}$$

with

$$x_0 = x(0) = \frac{1}{c},$$

so that

$$x(t) = \frac{1}{\int_0^t \frac{1}{K(\xi)} \exp(-\int_0^\xi \mu(\sigma) d\sigma) d\xi + \frac{1}{x_0}}$$

and using back-substitution of $J(t) = x(t)\, \exp(-\int_0^t \mu(\sigma)\, d\sigma)$ gives

$$\begin{aligned}
J(t) &= \frac{\exp(-\int_0^t \mu(\sigma) d\sigma)}{\int_0^t \frac{1}{K(\xi)} \exp(-\int_0^\xi \mu(\sigma) d\sigma) d\xi + \frac{1}{J_0}} \\
&= \frac{J_0 \exp(-\int_0^t \mu(\sigma) d\sigma)}{J_0 \int_0^t L(\xi) d\xi + 1}
\end{aligned}$$

with $L(\xi) = \frac{1}{K(\xi)} \exp(-\int_0^\xi \mu(\sigma) d\sigma)$.

S1.2 Time delay calculation

Before we run any simulations, we first calculate development times for immature life stages ($\tau_{E_t}, \tau_{J_t}, \tau_{I_t}$) and the extrinsic incubation period (EIP_t) for the temperatures given at all days of the year $t = 0 \ldots 365$. Starting from any given time point t_0, we sum over all the following inverse development times $\frac{1}{\tau_{X_t}}$, that denote the fraction of daily development, until this sum reaches 1:

$$\tau_X(t) = \inf\{n \in \mathbb{N}^+ : \sum_{t=t_0}^{n} \frac{1}{\tau_{X_t}} \geq 1\}.$$

For example, if for days $t = 1, 2, 3$ temperatures would drop slightly and the juvenile development times would be $\tau_J = 10, 12, 13$ days. We would calculate that after these three days, $\frac{1}{10} + \frac{1}{12} + \frac{1}{13} = 26\%$ of the development would be completed. We would continue to add inverse development times until we reach 100% development and take the final number of days as development period.

Note that this approach is independent of the step size chosen to solve the numeric equations. The approach by **(author?)** [1], in which the time delay itself is modelled by a differential equation, is dependent on the step size. If the step size is not chosen small enough, this could for example lead to unexpected differences in an incubation period before and after a spike in temperature.

S1.3 ODE vs. DDE dynamics

The extrinsic incubation period (EIP) is the period a mosquito spends infected but not yet infectious. During this time, the pathogen (the dengue virus in our case) has to replicate and spread in the mosquito body before its saliva gets infectious and the pathogen can be transmitted to a susceptible person during a bite. There are different ways to model the transition from infected to infectious mosquitoes, e.g. with ordinary differential equations (ODEs) or delayed differential equations (DDEs). Using ODEs or DDEs imposes different assumptions on the infection dynamics.

For this comparison, we will assume that we only have the host and no vector, i.e. infected hosts can directly infect susceptible hosts like influenza for example. The same principles are applicable to vector-host models though.

ODE model

SEI denotes susceptible, exposed, and infectious mosquitoes for the infection dynamics. In ODEs, the rate of change of the classes $\mathbf{u} = (S, E, I)$ depends on a reaction function f that describes a linear or non-linear relationship of these classes; $\frac{d}{dt}\mathbf{u}(t) = f(\mathbf{u}(t))$. The (simplified) equations are given by:

$$\begin{aligned}
\tfrac{d}{dt}S(t) &= -\beta S(t)I(t) - \mu S(t)\\
\tfrac{d}{dt}E(t) &= \beta S(t)I(t) - \gamma E(t) - \mu E(t)\\
\tfrac{d}{dt}I(t) &= \gamma E(t) - \mu E(t)
\end{aligned}$$

with β the infection probability, μ the mortality rate that is the same for all three classes, and γ the incubation rate, i.e. the inverse of the EIP, $\gamma = \frac{1}{\text{EIP}}$.

DDE model

Another approach to model this incubation period are delayed differential equations (DDEs). Here, the rate of change at a certain time not only depends on the current state but also on previous states at time $t - \tau$; $\frac{d}{dt}\mathbf{u}(t) = f(\mathbf{u}(t), \mathbf{u}(t - \tau))$. Equations are given by:

$$\begin{aligned}
\tfrac{d}{dt}S(t) &= -\beta S(t)I(t) - \mu S(t)\\
\tfrac{d}{dt}E(t) &= \beta S(t)I(t) - \beta S(t-\tau)I(t-\tau)\mathrm{e}^{-\int_{t-\tau}^{t}\mu d\sigma} - \mu E(t)\\
\tfrac{d}{dt}I(t) &= \beta S(t-\tau)I(t-\tau)\mathrm{e}^{-\int_{t-\tau}^{t}\mu d\sigma} - \mu I(t)
\end{aligned}$$

In this case, the time delay τ is the EIP. The transition of mosquitoes into class I does not start before the EIP is over. But each time step, a certain proportion of mosquitoes die. After the EIP has passed, all the mosquitoes that have not died in the meantime move to class I. The factor $\mathrm{e}^{-\int_{t-\tau}^{t}\mu d\sigma}$ denotes for this mortality experienced over the EIP. Note that the incidence rate βSI is written without the delay terms in the susceptible's equation. This is necessary as otherwise infected but not yet infectious individuals remained in the susceptible class for the period τ and could then get infected again. The total number of individuals are then given by $N := S + I + E$.

The DDE model could only be calculated with classes S and I but in our model, we need to calculate class E as the exposed females also lay eggs.

Comparison

The difference between the two model assumptions becomes clear when comparing single infection cases directly, see Figure A. After a single infection case is introduced from outside at day 0, e.g. through a mosquito bite, the first infectious individual in the ODE model appears at day 1 while it appears only after EIP = 10 days with the DDE model.

Using ODEs assumes that after the end of the EIP, 50% of exposed mosquitoes have become infectious and changed into class I. However: before and after the end of the EIP, there are constantly mosquitoes changing from class E to class I. The distribution of transitions is not bell shaped (the transitions are not normally distributed) but rather resembles an exponential decay which is problematic: Most mosquitoes change from class E to I after day 1, regardless of the EIP, and a tiny fraction will still wait to become infectious in 100 years. In contrast, using DDEs assumes that after the end of the EIP, the mosquitoes that do not die in the exposed state, become infectious all at once.

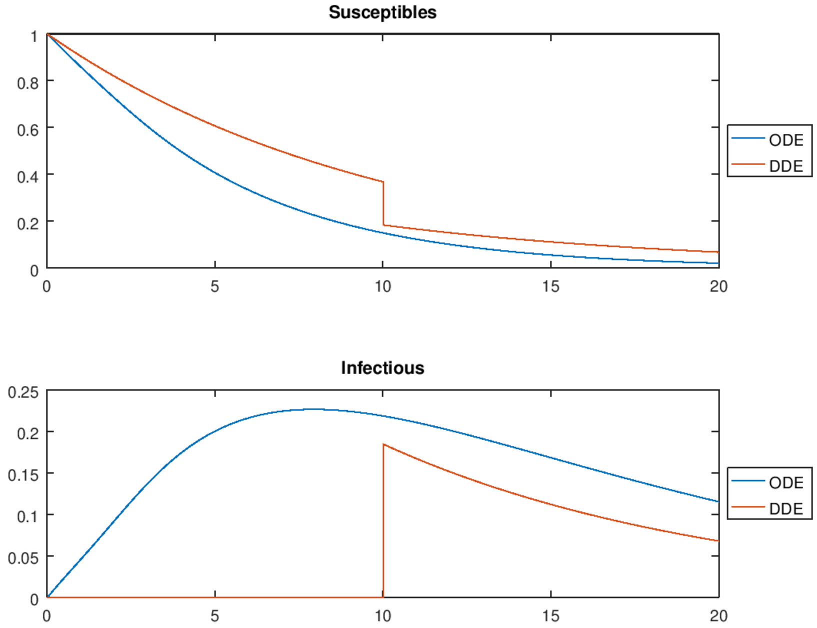

Figure A: Early disease onset. A susceptible meets an infected individual at time $t = 0$. In the DDE model, the number of infectious increases instantly, while it only after the duration of the EIP (10 days) in the DDE model. All individuals experience the same mortality in both models.

If τ is small, the models give similar results which is in accordance with **(author?)** [2]. The more the EIP or τ increases, the more the output of the ODE and DDE models will differ. Figure B shows the disease dynamics without mortalities for $\tau = 4$ and $\tau = 18$ days.

It is thus important to use DDE when analysing disease dynamics in which the EIP can take more than a week due to its temperature-dependence. Otherwise, the length and amplitude of disease outbreaks could be overestimated.

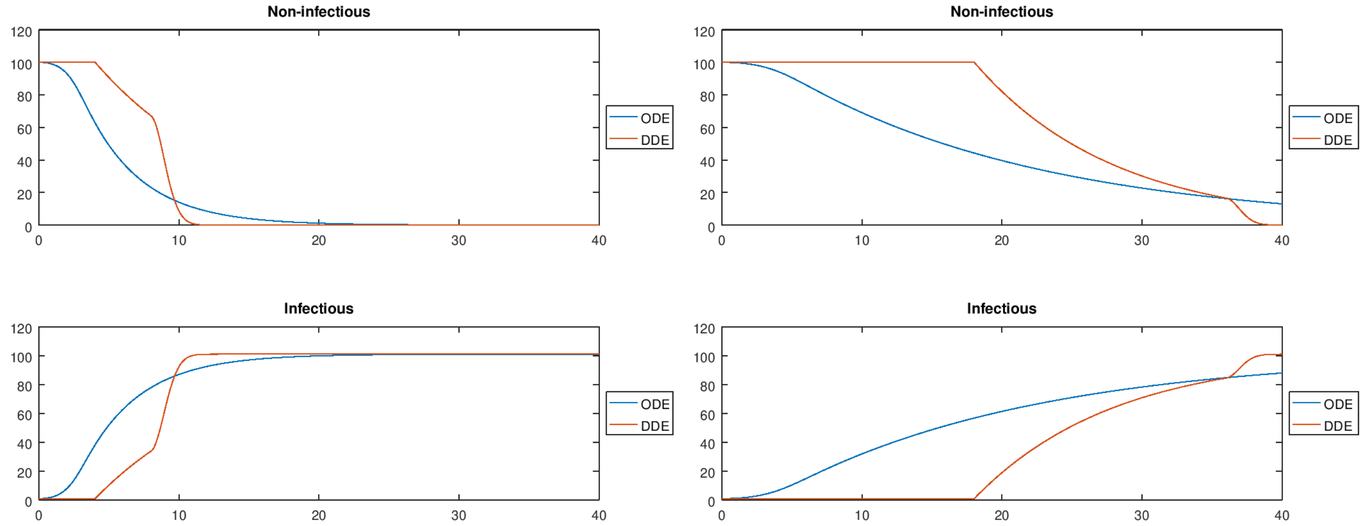

Figure B: Effect of EIP on models. Left: EIP = 4 days. Both ODE and DDE model show high infected numbers after about 10 days. Right: EIP = 18 days. While the DDE model does not show any infections in the first 18 days, the ODE model constantly increases. Both models show similar numbers only after day 30.

S1.4 Sensitivity Analysis

To investigate the influence of each parameter on the cumulative number of dengue cases in 2014, we perform the elementary effects test, EET [3], see Figure C. The EET measures the influence of single input parameters on model outputs, as well as their degree of interaction with other parameters. Latin hypercube sampling is used to vary parameters in the range of ±10% of the standard setting [4]. The model is then run with Guangzhou 2014 climate data until convergence is reached. The total number of cases at the end of the year is then retained as reference. Octave scripts for this method are derived from the SAFE toolbox [5].

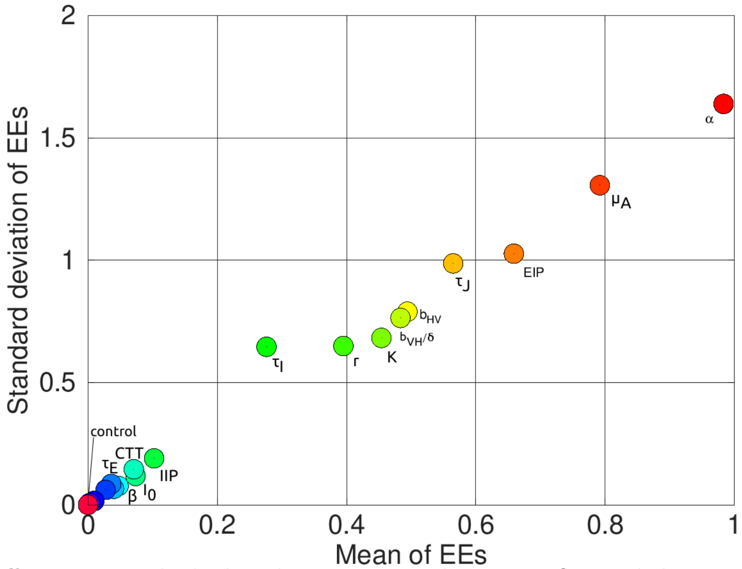

Figure C: Elementary effects test. The higher the mean EEs, the more influential the parameter on the modelled number of cases. The higher the standard deviation of the EEs, the larger its degree of interactions with other parameters.

The total number of dengue cases will be strongly associated with the basic reproduction number for vector-borne diseases:

$$R_0 = \sqrt{\frac{\alpha^2\ b_{HV}\ b_{VH}\ m}{r\ \mu_A\ (1 + \text{EIP}\ \mu_A)}} \quad (1)$$

This relationship is reflected by our sensitivity analysis: The biting rate, α, and the mosquito's adult mortality, μ_A, that appear twice in the formula, have the biggest effect on the simulated number of cumulative case, followed by the extrinsic incubation period, EIP, and transmission probabilities from host to vector, b_{HV}, and vector to host, b_{VH}. Factors that have a larger impact on the vector-to-host ratio m, including the environmental carrying capacity K, and development periods for juvenile and immature females, τ_J and τ_I, also have larger impacts. The other remaining factors have smaller impacts on m and the cumulative number of dengue cases. The distributions for mean and standard deviation of EEs indicate that those parameters that interact with other parameters also have a bigger effect on the model output.

S1.5 Pearl River Delta

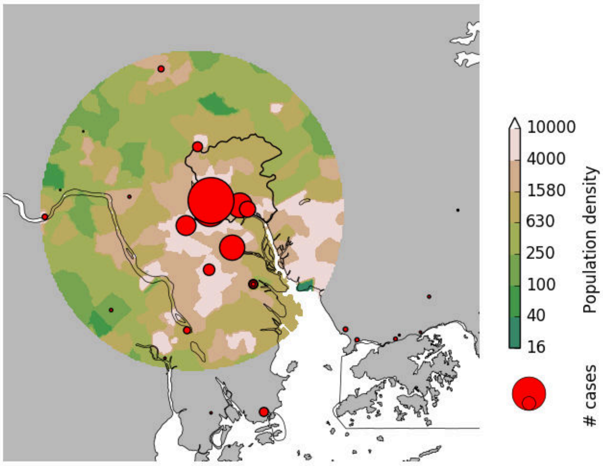

Figure D: Dengue cases in the Pearl River Delta 2014. While the biggest number of cases occurred within city limits of Guangzhou (thick black line), neighbouring cities and counties were responsible for about three quarter of the cases in this area. Maps created with Python package Basemap [6].

S1.6 Equilibria

In order to determine model equilibria, we first write down our full equation system:

$$
\begin{aligned}
\tfrac{d}{dt}E(t) &= \beta\,(1-\omega)\,A(t) - (\beta\,(1-\omega)\,A(t-\tau_E))\;e^{-\int_{t-\tau_E}^{t}\mu_E(\sigma)\,d\sigma} - \mu_E\,E(t)\\
\tfrac{d}{dt}J(t) &= \beta\,(1-\omega)\,A(t-\tau_E)\,e^{-\int_{t-\tau_E}^{t}\mu_E(\sigma)\,d\sigma} + \phi\,\gamma\,E_d(t)\\
&\quad - \frac{\left(\beta\,(1-\omega)\,A(t-\tau_E-\tau_J)\,e^{-\int_{t-\tau_E-\tau_J}^{t-\tau_J}\mu_E(\sigma)\,d\sigma} + \phi\,\gamma\,E_d(t-\tau_J)\right)\,e^{-\int_{t-\tau_J}^{t}\mu_J(\sigma)\,d\sigma)}}{1+\left(\beta\,(1-\omega)\,A(t-\tau_E-\tau_J)\,e^{-\int_{t-\tau_E-\tau_J}^{t-\tau_J}\mu_E(\sigma)\,d\sigma} + \phi\,\gamma\,E_d(t-\tau_J)\right)\int_{t-\tau_J}^{t}L(u)\,du} - \mu_J\,J(t) - \frac{J(t)^2}{K(t)}\\
\tfrac{d}{dt}I(t) &= 0.5\,\frac{\left(\beta\,(1-\omega)\,A(t-\tau_E-\tau_J)\,e^{-\int_{t-\tau_E-\tau_J}^{t-\tau_J}\mu_E(\sigma)\,d\sigma} + \phi\,\gamma\,E_d(t-\tau_J)\right)\,e^{-\int_{t-\tau_J}^{t}\mu_J(\sigma)\,d\sigma)}}{1+\left(\beta\,(1-\omega)\,A(t-\tau_E-\tau_J)\,e^{-\int_{t-\tau_E-\tau_J}^{t-\tau_J}\mu_E(\sigma)\,d\sigma} + \phi\,\gamma\,E_d(t-\tau_J)\right)\int_{t-\tau_J}^{t}L(u)\,du}\\
&\quad - 0.5\left(\frac{\left(\beta\,(1-\omega)\,A(t-\tau_E-\tau_J-\tau_I)\,e^{-\int_{t-\tau_E-\tau_J-\tau_I}^{t-\tau_J-\tau_I}\mu_E(\sigma)\,d\sigma} + \phi\,\gamma\,E_d(t-\tau_J-\tau_I)\right)\,e^{-\int_{t-\tau_J-\tau_I}^{t-\tau_I}\mu_J(\sigma)\,d\sigma}}{1+\left(\beta\,(1-\omega)\,A(t-\tau_E-\tau_J-\tau_I)\,e^{-\int_{t-\tau_E-\tau_J-\tau_I}^{t-\tau_J-\tau_I}\mu_E(\sigma)\,d\sigma} + \phi\,\gamma\,E_d(t-\tau_J-\tau_I)\right)\int_{t-\tau_J-\tau_I}^{t-\tau_I}L(u)\,du}\right)\\
&\quad \cdot e^{-\int_{t-\tau_I}^{t}\mu_A(\sigma)\,d\sigma} - \mu_A\,I(t)\\
\tfrac{d}{dt}A(t) &= 0.5\left(\frac{\left(\beta\,(1-\omega)\,A(t-\tau_E-\tau_J-\tau_I)\,e^{-\int_{t-\tau_E-\tau_J-\tau_I}^{t-\tau_J-\tau_I}\mu_E(\sigma)\,d\sigma} + \phi\,\gamma\,E_d(t-\tau_J-\tau_I)\right)\,e^{-\int_{t-\tau_J-\tau_I}^{t-\tau_I}\mu_J(\sigma)\,d\sigma}}{1+\left(\beta\,(1-\omega)\,A(t-\tau_E-\tau_J-\tau_I)\,e^{-\int_{t-\tau_E-\tau_J-\tau_I}^{t-\tau_J-\tau_I}\mu_E(\sigma)\,d\sigma} + \phi\,\gamma\,E_d(t-\tau_J-\tau_I)\right)\int_{t-\tau_J-\tau_I}^{t-\tau_I}L(u)\,du}\right)\\
&\quad \cdot e^{-\int_{t-\tau_I}^{t}\mu_A(\sigma)\,d\sigma} - \mu_A\,A(t)\\
\tfrac{d}{dt}E_d(t) &= \beta\,\omega\,A(t) - \phi\,E_d(t)
\end{aligned}
$$

We now assume that temperature and precipitation are constant and thus that all parameters are stable too: $\mu_E(T(t)) = \mu_E$, $\tau_E(T(t)) = \tau_E$, etc. We get:

$$
\begin{aligned}
\tfrac{d}{dt}E(t) &= \beta\,(1-\omega)\,A(t) - (\beta\,(1-\omega)\,A(t-\tau_E))\;e^{-\tau_E\,\mu_E} - \mu_E E(t)\\
\tfrac{d}{dt}J(t) &= \beta\,(1-\omega)\,A(t-\tau_E)\,e^{-\tau_E\,\mu_E} + \phi\,\gamma\,E_d(t)\\
&\quad - \frac{\left(\beta\,(1-\omega)\,A(t-\tau_E-\tau_J)\,e^{-\tau_E\,\mu_E} + \phi\,\gamma\,E_d(t-\tau_J)\right)e^{-\tau_J\mu_J}}{1+(\beta\,(1-\omega)\,A(t-\tau_E-\tau_J)\,e^{-\tau_E\,\mu_E} + \phi\,\gamma\,E_d(t-\tau_J))\,\frac{\tau_J}{K}e^{-\tau_J\mu_J}} - \mu_J J(t) - \frac{J(t)^2}{K}\\
\tfrac{d}{dt}I(t) &= 0.5\frac{\left(\beta\,(1-\omega)\,A(t-\tau_E-\tau_J)\,e^{-\tau_E\,\mu_E} + \phi\,\gamma\,E_d(t-\tau_J)\right)e^{-\tau_J\mu_J}}{1+(\beta\,(1-\omega)\,A(t-\tau_E-\tau_J)\,e^{-\tau_E\,\mu_E} + \phi\,\gamma\,E_d(t-\tau_J))\,\frac{\tau_J}{K}e^{-\tau_J\mu_J}}\\
&\quad - 0.5\frac{\left(\beta\,(1-\omega)\,A(t-\tau_E-\tau_J-\tau_I)\,e^{-\tau_I\,\mu_A} + \phi\,\gamma\,E_d(t-\tau_J-\tau_I)\right)e^{-\tau_J\mu_J}}{1+(\beta\,(1-\omega)\,A(t-\tau_E-\tau_J-\tau_I)\,e^{-\tau_I\,\mu_A} + \phi\,\gamma\,E_d(t-\tau_J-\tau_I))\,\frac{\tau_J}{K}e^{-\tau_J\mu_J}}e^{-\tau_I\,\mu_A} - \mu_A I(t)\\
\tfrac{d}{dt}A(t) &= 0.5\frac{\left(\beta\,(1-\omega)\,A(t-\tau_E-\tau_J-\tau_I)\,e^{-\tau_I\,\mu_A} + \phi\,\gamma\,E_d(t-\tau_J-\tau_I)\right)e^{-\tau_J\mu_J}}{1+(\beta\,(1-\omega)\,A(t-\tau_E-\tau_J-\tau_I)\,e^{-\tau_I\,\mu_A} + \phi\,\gamma\,E_d(t-\tau_J-\tau_I))\,\frac{\tau_J}{K}e^{-\tau_J\mu_J}}e^{-\tau_I\,\mu_A} - \mu_A A(t)\\
\tfrac{d}{dt}E_d(t) &= \beta\,\omega A(t) - \phi E_d(t).
\end{aligned}
$$

At equilibria DDEs behave like ODEs, as in the limit $x(t-\tau) = x(t)$. Thus we get:

$$
\begin{aligned}
\tfrac{d}{dt}E &= \beta\,(1-\omega)\,A\,(1-e^{-\tau_E\,\mu_E}) - \mu_E\,E\\
\tfrac{d}{dt}J &= \left(\beta\,(1-\omega)\,A\,e^{-\tau_E\,\mu_E} + \phi\,\gamma\,E_d\right)\left(1 - \frac{e^{-\tau_J\,\mu_J}}{1+(\beta\,(1-\omega)\,A\,e^{-\tau_E\,\mu_E} + \phi\,\gamma\,E_d)\,\frac{\tau_J}{K}e^{-\tau_J\,\mu_J}}\right) - \mu_J\,J - \frac{J^2}{K}\\
\tfrac{d}{dt}I &= 0.5\frac{\left(\beta\,(1-\omega)\,A\,e^{-\tau_E\,\mu_E} + \phi\,\gamma\,E_d\right)e^{-\tau_J\,\mu_J}}{1+(\beta\,(1-\omega)\,A\,e^{-\tau_E\,\mu_E} + \phi\,\gamma\,E_d)\,\frac{\tau_J}{K}e^{-\tau_J\,\mu_J}}(1-e^{-\tau_I\,\mu_A}) - \mu_A\,I\\
\tfrac{d}{dt}A &= 0.5\frac{\left(\beta\,(1-\omega)\,A\,e^{-\tau_I\,\mu_A} + \phi\,\gamma\,E_d\right)e^{-\tau_J\,\mu_J}}{1+(\beta\,(1-\omega)\,A\,e^{-\tau_I\,\mu_A} + \phi\,\gamma\,E_d)\,\frac{\tau_J}{K}e^{-\tau_J\,\mu_J}}e^{-\tau_I\,\mu_A} - \mu_A\,A\\
\tfrac{d}{dt}E_d &= \beta\,\omega\,A - \phi\,E_d
\end{aligned}
$$

Assuming E, J, I, A, E_d all positive, we get a trivial, $x_1^\star = 0$, and a non-trivial solution, $x_2^\star = (E^\star, J^\star, I^\star, A^\star, E_d^\star)$,

with

$$
\begin{aligned}
A^\star &= \frac{1}{2}\frac{K(\gamma\beta\omega e^{-\mu_J\tau_J-\mu_A\tau_I} + \beta(1-\omega)\, e^{-\mu_E\tau_E-\mu_J\tau_J-\mu_A\tau_I} - 2\mu_A)}{(\gamma\beta\omega + \beta(1-\omega)e^{-\mu_E\tau_E})\mu_A\tau_J e^{-\mu_J\tau_J}} \\
E_d^\star &= \frac{\beta\omega}{\phi}A^\star \\
E^\star &= \frac{\beta(1-\omega)(1-e^{-\mu_E\tau_E})}{\mu_E}A^\star
\end{aligned}
$$

and $I^\star$ and $J^\star$ being longer terms we do not write down.
It is now of interested for what parameter range either equilibrium is stable. If the trivial equilibrium is stable, the mosquito population will not be able to successfully reproduce in the long run.

Stability

We now analyse the stability of the trivial equilibrium. Let $x^\star$ be an equilibrium of equation

$$\frac{d}{dt}x(t) = f(x, x(t-\tau_1), \dots x(t-\tau_n)), \tag{2}$$

and let $\delta x(t)$ be the displacement from this equilibrium, assumed small. We assume f is smooth around $x^\star$ and thus has derivatives of all orders. Accordingly,

$$x(t) = x^\star + \delta x(t)$$

and

$$\frac{d}{dt}x(t) = \frac{d}{dt}\delta x(t) = f(x^\star + \delta x(t), x^\star + \delta x(t-\tau_1), \dots x^\star + \delta x(t-\tau_n)).$$

Linearisation, using a Taylor series, yields

$$\frac{d}{dt}\delta x(t) \approx J_0 \delta x(t) + J_{\tau_1}\delta x(t-\tau_1) + \dots J_{\tau_n}\delta x(t-\tau_n)$$

with J_0 being the Jacobian with respect to x, and J_{τ_i} being the Jacobians with respect to $x(t-\tau_i)$, evaluated at $x(t) = x(t-\tau_1) = \dots x(t-\tau_n) = x^\star$. Following **(author?)** [7], we now assume that equation (2) has an exponential solutions, so that we can write $\delta x(t) = Be^{\lambda t}$, and thus we get

$$
\begin{aligned}
\frac{d}{dt}\delta x(t) &= J_0 B e^{\lambda t} + J_{\tau_1} B e^{\lambda(t-\tau_1)} + \dots J_{\tau_n} B e^{\lambda(t-\tau_n)} \\
&= (J_0 + J_{\tau_1}e^{-\lambda\tau_1} + \dots J_{\tau_n}e^{-\lambda\tau_n})Be^{\lambda t}
\end{aligned}
$$

and finally our characteristic equation with I being the identity matrix

$$|J_0 + J_{\tau_1}e^{-\lambda\tau_1} + \dots J_{\tau_n}e^{-\lambda\tau_n} - \lambda I| = 0.$$

Calculating the Jacobian matrices at the zero equilibrium $x^\star = 0$ gives

$$J_0 = \begin{pmatrix} -\mu_E & & & \beta(1-\omega) & \\ & -\mu_J & & & \phi\gamma \\ & & -\mu_A & & \\ & & & -\mu_A & \\ & & & \beta\omega & -\phi \end{pmatrix}$$

for the normal Jacobian with respect to $x(t)$ and Jacobians J_{τ_E}, J_{τ_J}, $J_{\tau_E+\tau_J}$,... with respect to $x(t-\tau_E)$, $x(t-\tau_J)$, $x(t-\tau_E-\tau_J)$,..., respectively.

As all our DDEs in the fully written equation system only have delays for A and E_d, the remaining Jacobians J_{τ_E}, J_{τ_J}, $J_{\tau_E+\tau_J}$,... only have entries in the last two columns. Thus, the upper left 3×3 submatrix of the Jacobian combination will stay a diagonal matrix. We see that only entries in the bottom right 2×2 submatrix will affect the determinant of the Jacobians combination. These entries are $\frac{\partial A}{\partial A(t-\tau_E-\tau_J-\tau_I)}$ and $\frac{\partial A}{\partial E_d(t-\tau_J-\tau_I)}$, and assuming constant parameters, $\mu_E(T(t)) = \mu_E, \ldots$ gives:

$$\begin{aligned}
\frac{\partial A}{\partial A(t-\tau_E-\tau_J-\tau_I)}\Big|_{x=0} &= 0.5\,\beta\,(1-\omega)\,e^{-\int_{t-\tau_E-\tau_J-\tau_I}^{t-\tau_J-\tau_I}\mu_E(\sigma)d\sigma}e^{-\int_{t-\tau_J-\tau_I}^{t-\tau_I}\mu_J(\sigma)d\sigma}e^{-\int_{t-\tau_I}^{t}\mu_A(\sigma)d\sigma} \\
&= 0.5\,\beta\,(1-\omega)e^{-\tau_E\,\mu_E-\tau_J\mu_J-\tau_I\,\mu_A} \\
\frac{\partial A}{\partial E_d(t-\tau_J-\tau_I)}\Big|_{x=0} &= 0.5\,\phi\,\gamma\,e^{-\int_{t-\tau_J-\tau_I}^{t-\tau_I}\mu_J(\sigma)d\sigma}e^{-\int_{t-\tau_I}^{t}\mu_A(\sigma)d\sigma} \\
&= 0.5\,\phi\,\gamma\,e^{-\tau_J\mu_J-\tau_I\,\mu_A}.
\end{aligned}$$

For our characteristic equation, we get

$$|J_0 + e^{-\lambda(\tau_E+\tau_J+\tau_I)}J_{\tau_E+\tau_J+\tau_I} + e^{-\lambda(\tau_J+\tau_I)}J_{\tau_J+\tau_I} - \lambda I| = 0,$$

and calculating the determinant yields:

$$\begin{aligned}
(-\mu_E-\lambda)(-\mu_J-\lambda)(-\mu_A-\lambda)\Big[\Big(&-\mu_A + 0.5\,\beta\,(1-\omega)\,e^{-\tau_E\,\mu_E-\tau_J\,\mu_J-\tau_I\,\mu_A}\,e^{-\lambda(\tau_E+\tau_J+\tau_I)} \\
&-\lambda\Big)(-\phi-\lambda) - \beta\,\omega\,0.5\,\phi\,\gamma\,e^{\tau_J\mu_J+\tau_I\,\mu_A}\,e^{-\lambda(\tau_J+\tau_I)}\Big] = 0.
\end{aligned}$$

Thus

$$\begin{aligned}
&\lambda = -\mu_E \quad \vee \quad \lambda = -\mu_J \quad \vee \quad \lambda = -\mu_A \quad \vee \\
&0 = \Big(-\mu_A + 0.5\,\beta\,(1-\omega)\,e^{-\tau_E\,\mu_E-\tau_J\,\mu_J-\tau_I\,\mu_A}\,e^{-\lambda(\tau_E+\tau_J+\tau_I)} - \lambda\Big)\,(-\phi-\lambda) \\
&\qquad - \beta\,\omega\,0.5\,\phi\,\gamma\,e^{-\tau_J\,\mu_J-\tau_I\,\mu_A}\,e^{-\lambda(\tau_J+\tau_I)}
\end{aligned}$$

Substitution of $\lambda := u + iv$ and using Euler's formula gives two equations that show that λ can be real or complex, with either positive or negative real parts. We thus try to reduce the complexity and only look at the active mosquito season with $\omega = 0$ and $\phi = 0$ to calculate λ for our standard parameter set. We get:

$$0 = \Big(-\mu_A + 0.5\,\beta\,e^{-\tau_E\,\mu_E-\tau_J\,\mu_J-\tau_I\,\mu_A}\,e^{-\lambda\,(\tau_E+\tau_J+\tau_I)} - \lambda\Big)\,(-\phi-\lambda)$$

and thus

$$\lambda = 0$$
$$\vee$$
$$\lambda = 0.5\,\beta\,e^{-\tau_E\,(\mu_E-\mu_A)-\tau_J\,(\mu_J-\mu_A)-W(0.5\,\beta\,\tau_E+\tau_J+\tau_I)\,e^{-\tau_E\,(\mu_E-\mu_A)-\tau_J\,(\mu_J-\mu_A)}} - \mu_A,$$

with $W(x)$ denoting the Lambert-W function. Calculating λ with actual values for $\tau_E(T)$, $\mu_A(T)$ etc., shows if the trivial equilibrium is stable or unstable. In this case, the population reaches a non-trivial equilibrium for the current model setting when the constant temperature is higher than 16.5 °C and lower than 32.5 °C. Thus, the temperature of the active mosquito season (roughly April until end of September) should be between these two values for a successful establishment of *Ae. albopictus*.

Figure E shows model runs for 365 days with temperatures ranging from 10 to 35 °C. The modelled mosquito population is only stable or increasing within the calculated stable range between 16.5 °C and lower than 32.5 °C. Population numbers at temperatures below or above this range approximate zero.

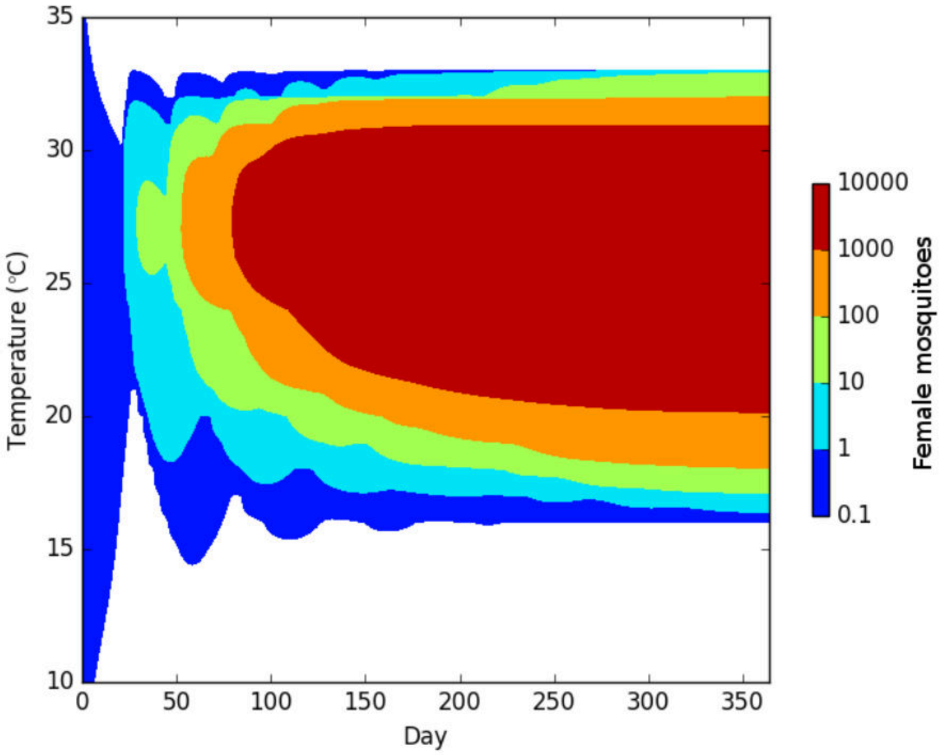

Figure E: Simulated adult female abundance as a function of temperature. The numbers of adult females are plotted over a period of 365 days for various temperatures, starting from 1 adult female. All numbers go down first as there are no eggs or larvae to replace dying females, before going up in the suitable range after 30 to 100 days.

S1.7 Updated suitability

Using the ROC plot indicates that using an index of 1 egg leading to 25 eggs after a year (instead of the used value of 1 egg leading to 1 egg in return) is giving a better fit to observation data, see Figure F. Here, the Taklamakan desert does not show up as suitable, while southern and eastern parts are still suitable.

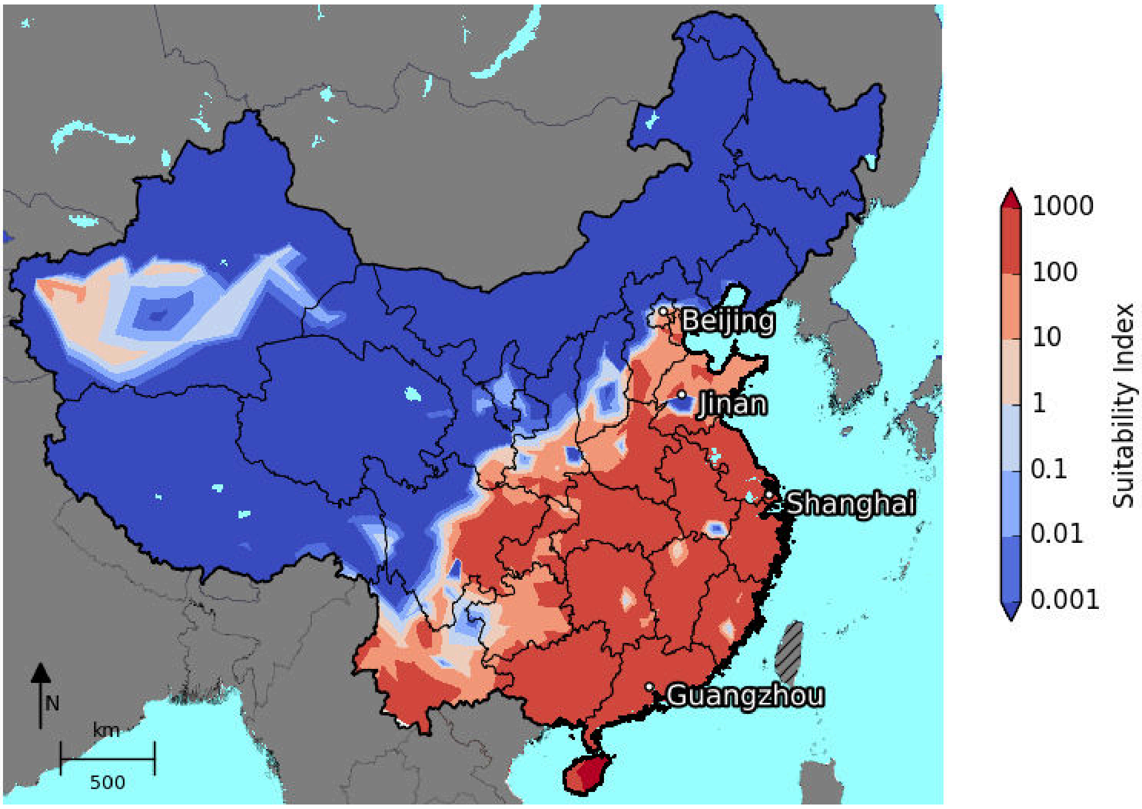

Figure F: China's suitability for *Ae. albopictus* with updated index. Modelled suitability index (E_0) for China for the period 2006 to 2016. Values above 400 indicate suitable regions, below unsuitable regions. The red line indicates the areas showing up as suitable with the original suitability index ($E_0 = 1$). The CMCD climate data set was used to drive the model. Maps created with Python package Basemap [6].

S1.8 China-wide BI data 2015

In addition to BI data for Guangzhou, monthly BI data for 21 out of 34 administrative divisions (provinces, autonomous regions, municipalities) were compared to model simulations for the year 2015. The model predicts the start of the mosquito season for April/May, lasting until September/October for northern, and November for southern provinces. The highest BI values are simulated in the provinces along the southern Chinese coast for the summer months and BI values decrease with latitude, see Figure G. Observations, however, show that BI values peak in more northern provinces, with BI>30 in Hebei (bordering Beijing), Shandong and Henan provinces in August. A correlation test gives Spearman's $\rho = 0.39$ ($p < 0.001$) for China-wide BI values. Rainfall data was absent for the provinces Jiangxi, Guizhou, and Tibet for 2014, so that mosquito numbers of 2013 were used to drive simulations for 2015 for these provinces.

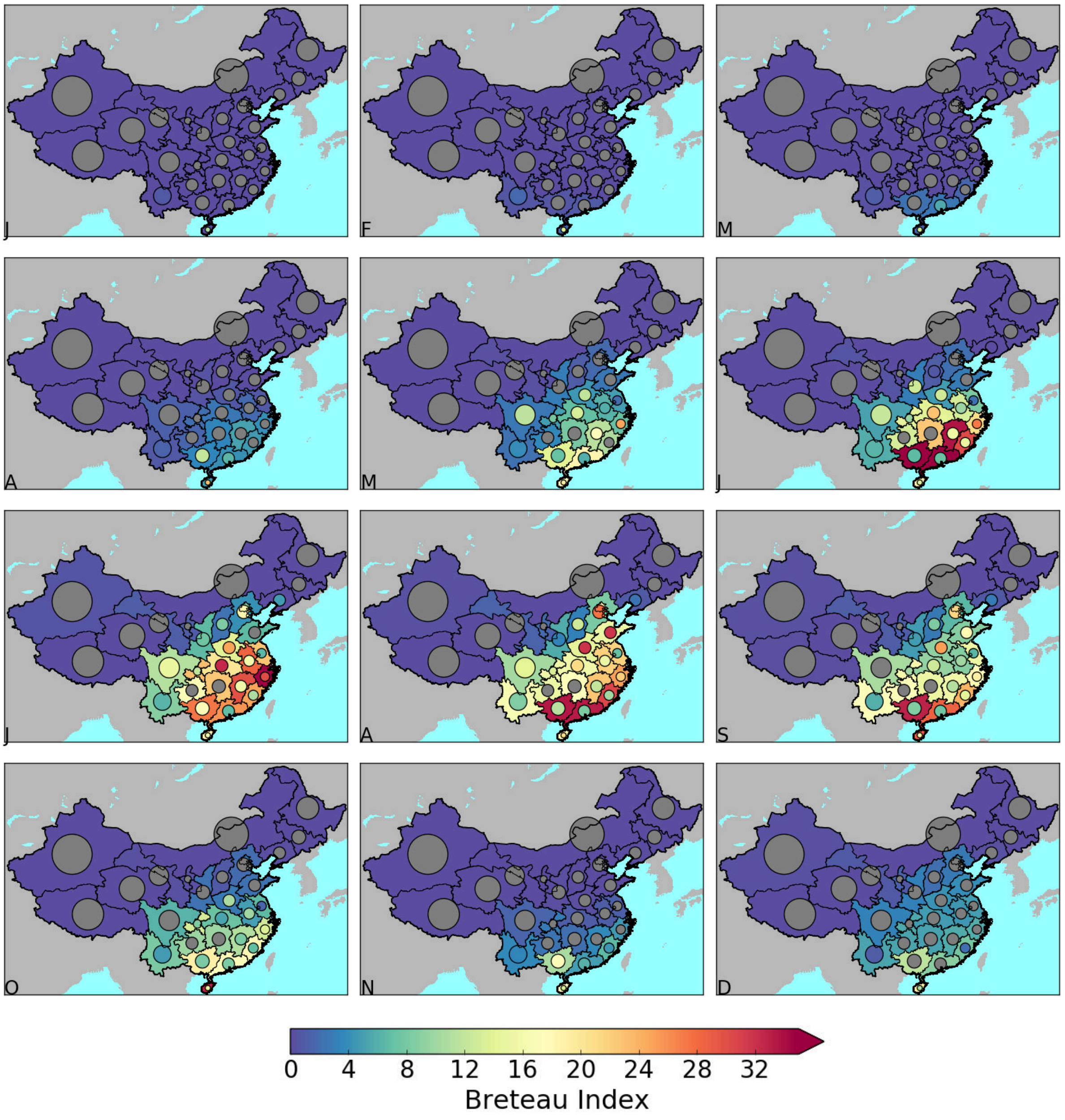

Figure G: Monthly BI values for 2015. Circle colour denotes observed BI, grey filling is used for missing values. Mean values of all data points included within province boundaries are used. Circle size relates to province size. Background colour of provinces denotes the simulated BI as the average of all weather station locations within province boundaries. A scaling factor of a=0.0001 was used for equation (13) due to the difference in human population densities between city and weather station locations. Maps created with Python package Basemap [6].

S1.9 DDE vs. ODE transmission risk

We repeated the simulations to calculate the potential length of the dengue transmission season using the equivalent ODE model (compare section S1.1) with identical parameters, see Figure H. In comparison to the DDE model, the ODE model predicts higher mosquito numbers and longer maximal season length. Predicted season length for all four cities would increase by approximately two months with the ODE model. More remarkable, though, is that the numbers of the ODE model are higher than the ones of the DDE model, the colder the climate is. This uneven augmenting of numbers by the ODE model shows the importance of incorporating more realistic mechanisms for vector and virus development, especially for temperate regions.

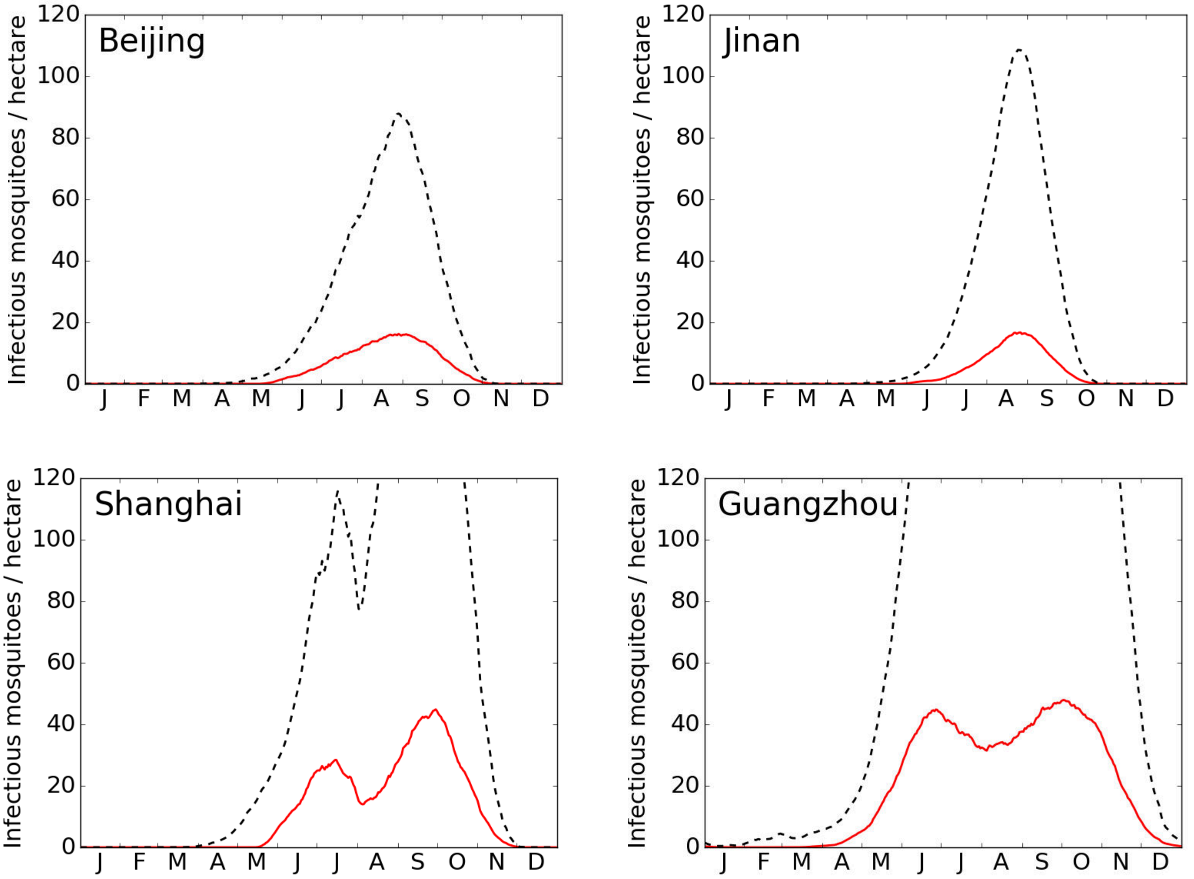

Figure H: DDEs vs. ODEs. Average densities of infectious female mosquitoes per hectare for 2006–2016. Red lines: DDE model calculations. Grey dashed lines: ODE model calculations. Identical parameters were used.

References

[1] Nisbet RM, Gurney WSC. The systematic formulation of population models for insects with dynamically varying instar duration. Theoretical Population Biology. 1983;23(1):114–135. doi:10.1016/0040-5809(83)90008-4.

[2] Kaddar A, Abta A, Alaoui HT. A comparison of delayed SIR and SEIR epidemic models. Nonlinear Analysis: Modelling and Control. 2011;16(2):181–190.

[3] Morris MD. Factorial Plans for Preliminary Sampling Computational Experiments. Technometrics. 1991;33(2):161–174. doi:10.1080/00401706.1991.10484804.

[4] McKay MD, Beckman RJ, Conover WJ. A comparison of three methods for selecting values of input variables in the analysis of output from a computer code. Technometrics. 2000;42(1):55–61. doi:10.1080/00401706.2000.10485979.

[5] Pianosi F, Sarrazin F, Wagener T. A Matlab toolbox for Global Sensitivity Analysis. Environmental Modelling and Software. 2015;70:80–85. doi:10.1016/j.envsoft.2015.04.009.

[6] Matplotlib Development Team. Python's Basemap Matplotlib Toolkit documentation. 2016;.

[7] Roussel M. Delay-differential equations; 2005. Available from: http://people.uleth.ca/roussel/nld/delay.pdf.
